# Genome and methylome of the oleaginous diatom *Cyclotella cryptica* reveal genetic flexibility toward a high lipid phenotype

**DOI:** 10.1186/s13068-016-0670-3

**Published:** 2016-11-25

**Authors:** Jesse C. Traller, Shawn J. Cokus, David A. Lopez, Olga Gaidarenko, Sarah R. Smith, John P. McCrow, Sean D. Gallaher, Sheila Podell, Michael Thompson, Orna Cook, Marco Morselli, Artur Jaroszewicz, Eric E. Allen, Andrew E. Allen, Sabeeha S. Merchant, Matteo Pellegrini, Mark Hildebrand

**Affiliations:** 1 Scripps Institution of Oceanography, University California San Diego, 9500 Gilman Drive, La Jolla, CA 92093-0202 USA; 2 Institute for Genomics and Proteomics, University of California, Los Angeles, CA 90095 USA; 3 J. Craig Venter Institute, 4120 Capricorn Lane, La Jolla, CA 92037 USA; 4 Department of Chemistry and Biochemistry, University of California, Los Angeles, CA 90095 USA

**Keywords:** Diatom, Genome sequence, *Cyclotella cryptica*, Algae biofuel, Carbon metabolism, DNA methylation

## Abstract

**Background:**

Improvement in the performance of eukaryotic microalgae for biofuel and bioproduct production is largely dependent on characterization of metabolic mechanisms within the cell. The marine diatom *Cyclotella cryptica,* which was originally identified in the Aquatic Species Program, is a promising strain of microalgae for large-scale production of biofuel and bioproducts, such as omega-3 fatty acids.

**Results:**

We sequenced the nuclear genome and methylome of this oleaginous diatom to identify the genetic traits that enable substantial accumulation of triacylglycerol. The genome is comprised of highly methylated repetitive sequence, which does not significantly change under silicon starved lipid induction, and data further suggests the primary role of DNA methylation is to suppress DNA transposition. Annotation of pivotal glycolytic, lipid metabolism, and carbohydrate degradation processes reveal an expanded enzyme repertoire in *C. cryptica* that would allow for an increased metabolic capacity toward triacylglycerol production. Identification of previously unidentified genes, including those involved in carbon transport and chitin metabolism, provide potential targets for genetic manipulation of carbon flux to further increase its lipid phenotype. New genetic tools were developed, bringing this organism on a par with other microalgae in terms of genetic manipulation and characterization approaches.

**Conclusions:**

Functional annotation and detailed cross-species comparison of key carbon rich processes in *C. cryptica* highlights the importance of enzymatic subcellular compartmentation for regulation of carbon flux, which is often overlooked in photosynthetic microeukaryotes. The availability of the genome sequence, as well as advanced genetic manipulation tools enable further development of this organism for deployment in large-scale production systems.

**Electronic supplementary material:**

The online version of this article (doi:10.1186/s13068-016-0670-3) contains supplementary material, which is available to authorized users.

## Background

Global environmental changes are happening at an increasingly rapid rate, and development of technologies to alleviate negative outcomes is urgently needed. One proposed solution is producing energy from biofuel, which is renewable and has fewer detrimental effects than the use of fossil fuels. A potential feedstock for biofuel production, microalgae, has garnered interest because of its high productivity. The overall requirement for land to grow algae to sustain the United States’ fuel supply is predicted to be relatively small (<4% of the total land mass) compared to other crops [1].

Developing technologies have increased the promise of algal biofuel to meet energy needs [2, 3]. These new advances were preceded by the US Department of Energy funded Aquatic Species Program (ASP) which produced pioneering work in aquaculture and large-scale production of biodiesel from microalgae [4]. During the ASP, algal species across the tree of life were assessed for their ability to accumulate abundant triacylglycerol (a precursor to biofuel), to grow under variable environmental conditions such as pH, salinity and temperature, and to grow at a productive and sustainable rate in outdoor raceway ponds [4]. Several genera stood out from the rest, including chlorophytes, chrysophytes, and diatoms. Diatoms (Bacillariophyta), which are naturally highly productive, accounted for ~60% of the top-performing species in a recommended list of biofuel production organisms produced by the ASP [4].

**Fig. 1:**
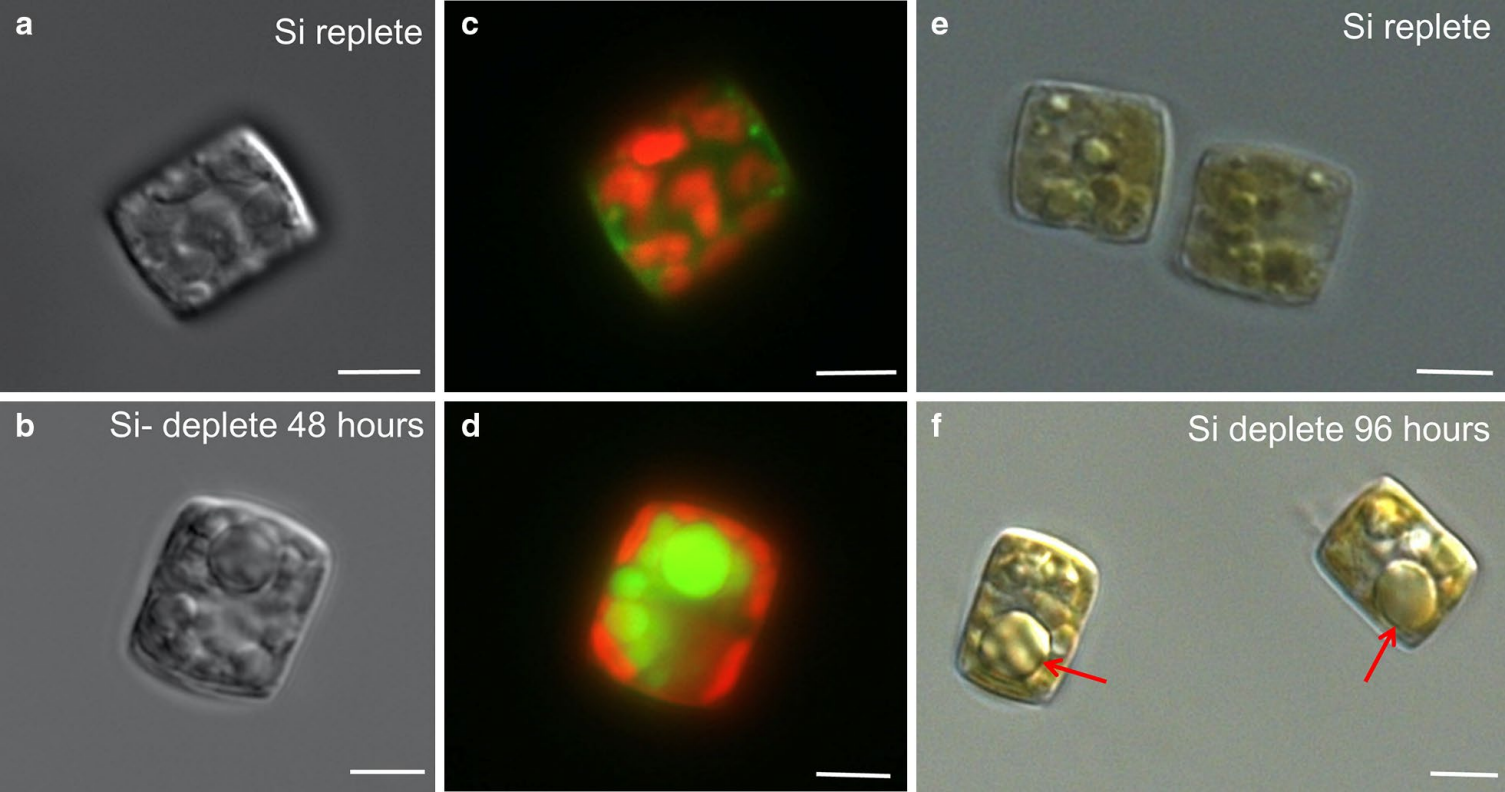
Lipid accumulation in *Cyclotella cryptica* under silicon deprivation. Grayscale image of *C. cryptica* in (**a**) silicon replete medium, 0 h lipid uninduced, or (**b**) 48 h silicon deplete, lipid induced. **c**, **d** Respective composite images of chlorophyll autofluorescence (*red*) and fluorescent lipophilic dye BODIPY (*green*). **e**, **f** Differential interference contrast image of silicon replete (**e**) and 96 h silicon deplete, lipid induced, with *red arrows* identifying lipid droplets (**f**). *Scale bars* 5 μm

*Cyclotella cryptica* (Fig. 1), a brackish water diatom isolated from Martha’s Vineyard, Massachusetts, was identified in the ASP as a top species for large-scale biofuel production. *Cyclotella cryptica* has been shown to be an excellent accumulator of lipid ([5, 6]; Fig. 1), is euryhaline, enabling flexibility in cultivation conditions [5–7], and can grow outdoors at levels between 20.0 and 29.7 g Ash Free Dry Weight (AFDW) m^2^/day in a 2.8 and 48 m^2^ pond, respectively [8, 9]. During the ASP, *C. cryptica* was used to investigate the lipid accumulation response during starvation for silicon, a macronutrient required by diatoms to synthesize their silicified cell walls, as well as to understand the properties of key enzymes involved in flux of carbon into lipid [10, 11]. More recently, based on a survey of 175 different microalgal strains, *C. cryptica* was selected as a top candidate for omega-3 fatty acid production, which is highly desirable for pharmaceutical and aquaculture applications, as well as a top producer of protein and nitrogen, suitable for agricultural feed [5]. In addition to having traits suited for commercial production, *C. cryptica* was the first chlorophyll-*c* containing algae to undergo stable nuclear transformation, a milestone in algal genetic engineering [12]. That study was the first of many significant advances in diatom genetic engineering, including determining the subcellular localization of proteins using GFP fusions, RNAi and antisense knockdowns, CRISPR, transcription activator-like effector nucleases (TALEN), and plasmid delivery via conjugation using an artificial episome [13–18].

Although *C. cryptica* and other microalgal species have excellent native productivity characteristics, cost analyses [19] indicate that further improvements are necessary to make algal biofuel production economically competitive with fossil fuels. Genetically based approaches are required to establish robust improved-productivity phenotypes. Both random mutagenesis and directed genetic manipulation can be used to accomplish this. The latter requires knowledge of an organism’s genome sequence, and such information has successfully been used to identify targets for genetic manipulation in diatoms to improve lipid productivity [14, 17, 20, 21]. To identify appropriate gene targets for manipulation, a thorough understanding of the enzymes involved in central carbon metabolism is required, including the number of isozymes that catalyze each chemical reaction and the compartment-specific localization of enzymes or enzymatic processes within the cell. It is especially important to consider organellar compartmentation of metabolic processes in a diatom cell, which, because of diatoms’ secondary endosymbiotic origin, contain additional compartments relative to the green algae. This includes the periplastid compartment surrounding the chloroplast and endoplasmic reticulum that also surrounds the periplastid compartment around the chloroplast [22–24]. Studying the diversity of core processes in carbon metabolism, such as glycolysis and fatty acid biosynthesis, within different lineages of algae [25, 26] as well as more closely within species of the same lineage [27–29], may allow researchers to address why certain species are better suited for biofuel production than others. Differences in the organization of primary carbon metabolism between species likely reflect differences in efficiencies in processing carbon, which relates to factors controlling their productivity. Understanding the dissimilarities amongst microalgae will help elucidate what constitutes an optimized biofuel/bioproduct production system, and enable a production species to be manipulated to create the desired product in the most efficient manner. The availability of additional algal genome sequences has enabled a more thorough comparison of these diverse polyphyletic organisms to identify metabolic steps that may influence the organism’s productivity characteristics. These comparisons are also essential to understanding the complex evolutionary history and ecology of microalgae.

To bring the promising characteristics of *C. cryptica* in line with current day approaches to improve productivity, we sequenced the nuclear, chloroplast, and mitochondrial genomes, performed a detailed in silico analysis of the core metabolic processes involved in or competing with lipid production and compared these to other diatom genomes, in particular the closely related *Thalassiosira pseudonana*. To investigate whether epigenetic factors influence lipid accumulation or primary metabolism in general, bisulfite sequencing was performed to examine the methylome of cells under silicon replete and silicon-deplete, lipid induced conditions. We further developed *C. cryptica* by application of genetic tools including fluorescent protein tagging and the use of an inducible promoter using genetic constructs derived from *T. pseudonana*. Genome sequence and data generated from this analysis provides a foundation to further improve this species for large-scale biofuel/bioproducts production and provide insight into central carbon metabolism in diatoms.

## Results and discussion

### Genome sequence determination and assembly

Three libraries with different average insert lengths were prepared from purified *C. cryptica* genomic DNA (Additional file 1: Additional methods). These were sequenced as paired-end 76-mer + 76-mer reads on an Illumina GA-IIx 120-tiles/lane run. Two genomic DNA mate pair libraries (aiming for 10 K nucleotide effective inserts) were prepared and run by Illumina service on a 48-tile/lane v3 HiSeq flow cell. The paired-end and mate pair libraries contributed ~23.4 G nucleotides (nt) and ~57.4 G nt, respectively, for a total of ~80.8 G nt. The main genome assembly was performed with an ABySS 1.3.1 single end, paired-end, mate-pair pipeline. There were 116,817 genomic contigs and the N50 value was 11,951 bp (Additional file 1: Figure S1).

In addition to the genome assembly, transcriptomes (13 samples) were generated for *C. cryptica* using RNAseq data under silicon limitation and nitrogen limitation (Additional file 1: Additional methods). Estimates of mRNA abundance were calculated for each gene model in terms of fragments per kbp of transcript per million mapped reads (FPKMs) to identify relative transcript levels for all genes across all experimental conditions. Gene expression patterns will be analyzed in a subsequent study.

### Genome statistics and gene model prediction

The estimated size of the haploid genome of *C. cryptica* was 161.7 Mbp, which is substantially larger than the 31 Mbp genome of *T. pseudonana*, a closely related model centric diatom. Similar to *T. pseudonana, C. cryptica* primarily exists in its vegetative state as diploid, and rarely undergoes sexual reproduction in controlled culture conditions. Multiple gene model prediction pipelines, including AUGUSTUS and MAKER, were evaluated to estimate the number of genes (Additional file 1: Table S1, Additional methods). The quality of predicted gene models was assessed by comparing *C. cryptica* RNAseq data and against manually curated *T. pseudonana* (version 3) gene models, which are supported by RNAseq and EST data [30–32]. MAKER predicted the fewest genes (Table 1; Additional file 1: Table S1), yet most of these models were supported by transcript data, and therefore, considered high confidence. However, intron and exon boundaries were improved in gene models from AUGUSTUS trained on the de novo *C. cryptica* RNA assembly. Despite these improvements, AUGUSTUS predicted a large proportion (30%) of fragmented, short gene models unsupported by transcript data (sum FPKM = 0.00, Table 1; Additional file 1: Table S1), which were presumed misscalls and removed from subsequent analysis. To leverage the strengths of the different predictors (boundary accuracy vs. expression-supported models), final ‘high-confidence’ gene models for *C. cryptica* are the set of AUGUSTUS gene models overlapping a MAKER prediction and assigned non-zero FPKM values (8133 genes). The total number of genes in *C. cryptica* per haploid genome, including the high confidence gene subset and all other AUGUSTUS models with RNAseq support was 21,121.

**Table 1:** Genomic features in *Cyclotella cryptica* and *Thalassiosira pseudonana*

Statistics	*C. cryptica*		*T. pseudonana*
Cell size	8 × 10 μm		4 × 5 μm
Nuclear genome size	161.7 Mbp (GC 43%)		31 Mbp (GC 47%)
Repeatome (%)	53		2
Classified repeats (%)	13		ND
Unclassified repeats (%)	40		ND

Gene models	High confidence	AUGUSTUS models	*T. pseudonana *Joint Genome Institute models
Percent coding DNA	10.0	19.2	66.3
Gene density (genes/Mbp)	50	131	379
Total models	8133	21,121	11,776
Average model length (bp)	1986	1471	1746
Average number of exons per gene	2.95	2.18	2.54
Average exon length (bp)	599	608	613
Average number of introns per gene	1.95	1.18	1.5
Average intron length (bp)	115	125	125
Chloroplast genome size (bp)	129,320		128,813
Total chloroplast models	132		127
Mitochondrial genome size (bp)	58,021		43,827
Total mitochondrial models	35		35

*T. pseudonana* data from [30, 34]

Gene density in *C. cryptica* was 3.4× lower than the closely related model species *T. pseudonana* with 19% coding DNA compared to 66% (Table 1). While the total number of gene models in *C. cryptica* was nearly double that of *T. pseudonana*, it did not scale with the increase in genome size; the *C. cryptica* genome was 5.2× larger than *T. pseudonana*. This generally correlated with the logarithmic relationship between gene content and genome size in microeukaryotes as shown in [33]. The low gene density in *C. cryptica* can be attributed to a large quantity of repeat sequence, 53% of the genome. The majority of repeat sequence was detected using RepeatModeler, a program to identify de novo repeat families. RepeatModeler predicted 314,059 unclassified repeat elements totaling to 40% of the genome (Additional file 1: Table S2b). In addition to novel repeat families, 8.6% of the genome is comprised of long terminal repeat elements, characteristic of Class I retrotransposons (Additional file 1: Tables S2a, S2b), which are common to diatoms and are hypothesized to contribute to the diversity and ecological success of these organisms in the oceans [31, 34].

### *Cyclotella cryptica* gene model repertoire

**Fig. 2:**
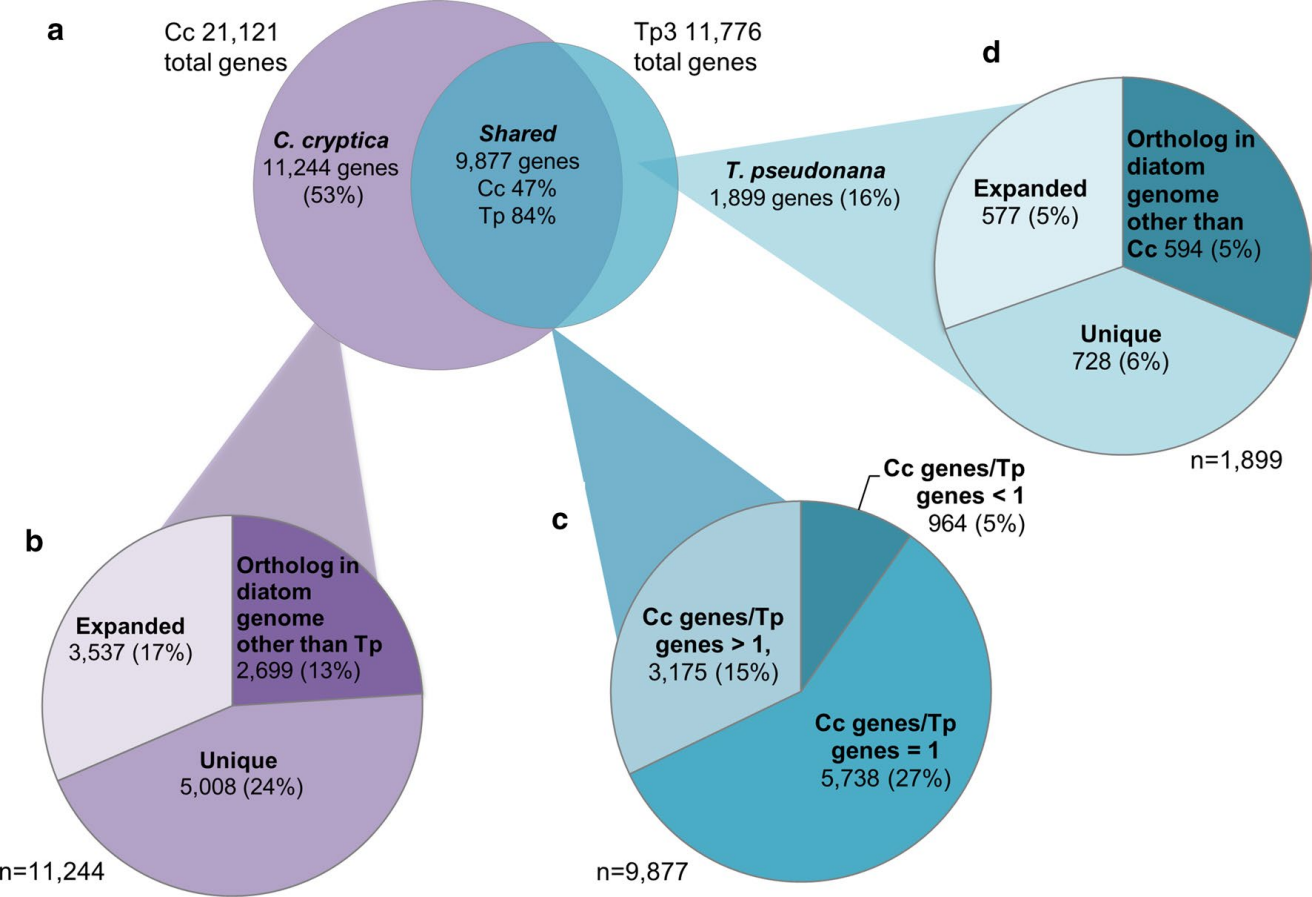
*Cyclotella cryptica* genetic components. Percentages reflect group relative to the total number of gene models in the species. **a** Comparison of genes between *C. cryptica* and *T. pseudonana* (JGI version 3). Over half the genes in *C. cryptica* (Cc) are not found in *T. pseudonana* (Tp). **b** Classification of *C. cryptica* specific genes (*n* = 11,244) relative to other diatom genomes. **c** Relative copy numbers of genes comparing *C. cryptica* and *T. pseudonana* (*n* = 9877). Percentages shown are based on the Cc total gene models. **d** Classification of *T. pseudonana* specific genes relative to other diatom genomes (*n* = 1899)

To determine the underlying basis for *C. cryptica’s* genetic characteristics relative to other diatoms, we defined orthologs and compared molecular divergence between *C. cryptica* and five other diatoms based on OrthoMCL, 18S phylogeny, and reciprocal best BLAST hit (RBH) analysis (Fig. 2; Additional file 1: Figure S2). *Thalassiosira pseudonana* and *C. cryptica* were the most closely related centric diatoms based on 18S and percent identity of RBH pairs, with 5498 shared pairs between them and 63.6% average percent identity across all RBH pairs (Additional file 1: Figure S2).

OrthoMCL uses a Markov Cluster algorithm to cluster putative orthologs across species and paralogs within species and is a powerful tool for comparative genomics and functional genome annotation [35]. Just under half of the genes have an orthologous match to *T. pseudonana* (9877 genes, 47%, Fig. 2a). By comparing all diatom genomes, we found 5008 genes (24% of total genes) were only found in *C. cryptica*, while 2699 genes (13%) shared a match to at least one other diatom but were absent in *T. pseudonana,* presumably due to gene loss (Fig. 2a, b). In addition, 17% of genes in *C. cryptica* are expanded orthologs to a gene(s) in *T. pseudonana*, which have resulted from either paralogous duplication or horizontal gene transfer in *C. cryptica,* or gene loss in *T. pseudonana* (Fig. 2b). 5738 genes were found in orthologous clusters where a gene in *C. cryptica* was present in equal copy numbers to genes in *T. pseudonana,* accounting for 27% of the total genes in *C. cryptica* (Fig. 2a, c). Based on the OrthoMCL data with these diatom genomes, the increase in gene content in *C. cryptica* relative to *T. pseudonana* is predominantly composed of unique genes not found in any other species, comprising 24% of the gene content. The proportion of unique genes in *T. pseudonana* was significantly less at 6% (Fig. 2b, d).

Genes unique to *C. cryptica* and not detected in the other diatoms contained minimal functional annotation based on KEGG, KOG, GO, Pfam, and TIGRfam; however, there was a notable enrichment in genes related to transposon processes, such as transposable element domains integrase (Pfam accession PF00665, 33 predicted genes), plant transposon gene (PF04827, 12 predicted genes), and reverse transcriptase (PF07727, 71 predicted genes). In contrast, the *T. pseudonana* genome contained only four genes with Integrase annotation, and one reverse transcriptase. These domains are indicators of mobile DNA elements, which are often responsible for genome size expansion. Given that *C. cryptica* and *T. pseudonana* have speciated relatively recently [36], the large amount of repetitive sequence with additional unique genes with transposable element annotation suggest that either *C. cryptica* has undergone a recent dramatic genome expansion event, more than doubling the amount of DNA and/or *T. pseudonana* has undergone a substantial genome deletion of repetitive sequence. The former scenario is favored due to the high number of *C. cryptica* unique genes with RNAseq support (Fig. 2b).

In addition to genes encoding mobile DNA element proteins, which were more abundant than those found in all other diatoms we analyzed, there was also an expansion of genes encoding proteins putatively involved in cell adhesion, signaling, and transport. Orthologous cluster (clust_77) contained 85 genes in *C. cryptica* with an enrichment of the discoidin domain, which is a glycoprotein putatively involved in carbohydrate binding and cytoskeletal organization [37]. In addition, relative to *T. pseudonana*, *C. cryptica* has two additional silicon transporters (clust_209, six copies total, including one partial sequence), and additional ATPases (clust_43, 67 copies) and phosphate and nitrate transporters (clust_248-NRT, Additional file 2). Since the *C. cryptica* cell is approximately twice as large in volume as *T. pseudonana*, it is reasonable to suggest that *C. cryptica* requires additional proteins at the cellular surface to compensate for the lower surface to volume ratio. Another possibility is that the expanded gene repertoire could allow for diversification function and metabolite flux. In addition to a nitrate transporter, *C. cryptica* possesses an additional plastid nitrate/formate transporter (g21971.t1), which may explain its high capacity for nitrogen assimilation, as shown in [5].

In previous studies, it has been demonstrated that up to 5% of diatom genes were proposed to arise via horizontal gene transfer [31]. We used the bioinformatic tool DarkHorse [38] to evaluate the contribution of horizontal gene transfer to gene expansion in *C. cryptica* (Additional file 2, DarkHorse). There were 16 unique genes of unknown function but matching viral sequences, however, most of these genes contained low levels of transcript and were not full length. Further analysis identified 312 genes (1.47% of total genes) with best matches to bacterial sequences, with 137 of those specific for Proteobacteria and 20 of cyanobacterial origin, a similar taxonomic grouping as found in [31]. Among these, there was an enrichment in genes belonging in the KOG class ‘Secondary metabolites biosynthesis, transport and catabolism’, with 6 of 312 (1.92%) versus 69 of 21,121 (0.33%) overall. In addition to horizontally acquired genes identified by DarkHorse, many of the predicted proteins contained no matches to any previously sequenced organism (4175, 20% of genes), which suggest that they were either acquired from an uncharacterized organism, have rapidly evolved in *C. cryptica*, or this diatom lineage is significantly unrepresented in sequencing databases. While it is possible that some of these unique genes may be erroneously predicted, several proteins did contain at least one Pfam domain. Overall, the number of genes acquired horizontally appears to be similar to that found in previous studies in diatoms with smaller genome sizes, and the increase in gene number in *C. cryptica* is primarily due to the prevalence of unknown genes, similar to findings from OrthoMCL (Fig. 2b; Additional file 2). While the annotation of these genes is limited, given the high number of repetitive sequence and transposable elements, we hypothesize that many of these unique genes are cryptic ORFs created from transposition events, similar to what has been detected in higher plants [39, 40]. It must be noted that many candidate foreign genes identified using the DarkHorse analysis may also be ancestral genes of diatoms, acquired vertically or via endosymbiosis. Examples of these are genes matching to organisms within ‘SAR excluding diatoms’ (the subgroup containing Stramenopiles, Alveolates, and Rhizaria) and Excavata; these genes are found in *C. cryptica* but appear to have been lost in the other diatoms included in this analysis.

### Methylation of the genome

DNA methylation can play a role in altering genome size and content by silencing genomic regions, and can have more dynamic effects in regulating expression of particular genes. The significant difference in genome size between *C. cryptica* with *T. pseudonana* and high levels of repetitive sequence prompted us to ask whether DNA methylation played a role in gene regulation and silencing of mobile DNA elements. Data from the pennate diatom *Phaeodactylum tricornutum* indicated that DNA methylation profiles correlate with transcript levels in some genes controlling nitrogen metabolism [41], therefore, an examination of dynamic changes in methylation in *C. cryptica* was also undertaken. To determine the relationship between DNA methylation and silicon limitation, which, similar to nitrogen, is an essential macronutrient to diatoms, we performed whole genome bisulfite sequencing on cells under silicon replete conditions (0 h), and after 48-h silicon depletion. In addition to silicon-limitation associated phenomena, a dramatic induction of triacylglycerol (TAG) levels occurs after 48 h of starvation (Fig. 1b, d; [4, 42]).

**Fig. 3:**
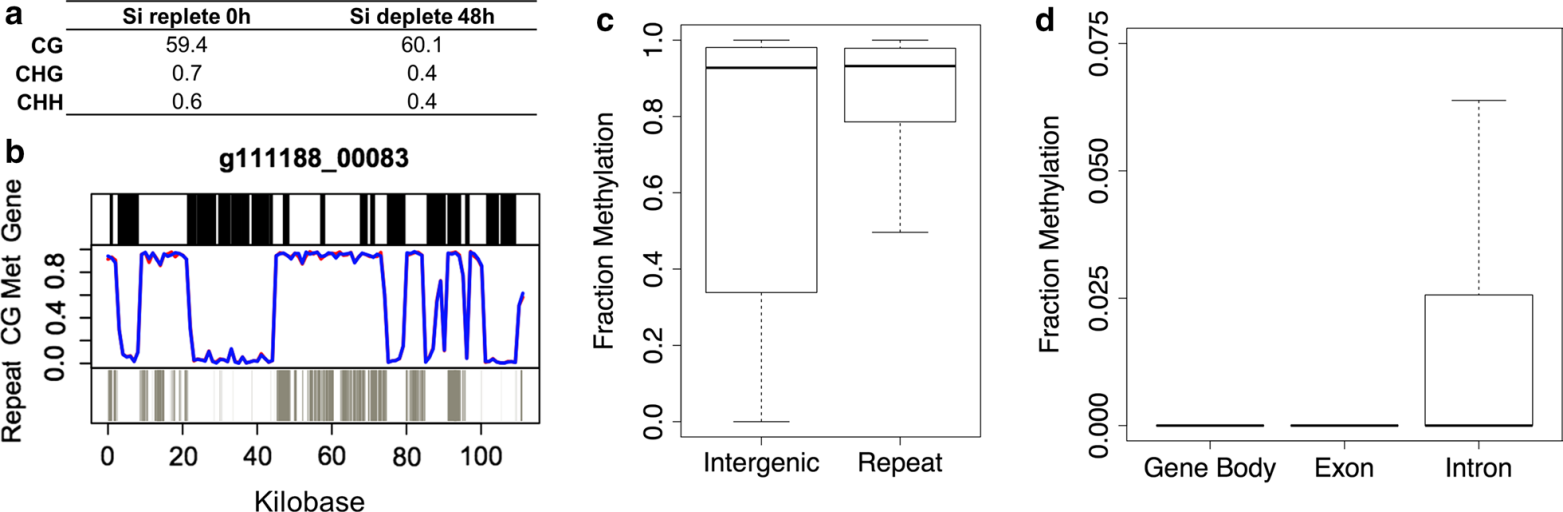
DNA methylation in *Cyclotella cryptica.* **a** Percent genomic methylation under both conditions according to cytosine motif (denoted at* left*, H = A, T, or C nucleotides). **b** Fraction methylation across genomic contig g111188_00083. *Blue* silicon replete, *red* silicon deplete 48 h. Augustus V3 models depicted in *black*, Repeat Modeler data depicted in *gray*. **c** Fraction methylation across intergenic, repeat, and **d** gene body, exon, intron regions. Outliers in **c** and **d** have been removed

The global per-cytosine (CG, CHH, CHG motifs, where H is any nucleotide but G) methylation level averaged in both conditions of the *C. cryptica* genome was 61% (Fig. 3a), the highest amount of DNA methylation in a diatom genome observed to date. Under both experimental conditions, methylation of cytosine sites was remarkably similar and bimodal (Additional file 1: Figure S3), suggesting that *C. cryptica* does not undergo substantial temporal shifts in overall methylation.

We found that in silicon replete, lipid uninduced (0 h) and 48 h silicon depletion, lipid induced conditions, methylation patterns across the whole genome were almost identical, with 98.7% correlation between the two conditions (Additional file 1: Figure S4). While [34, 41] detected shifts in DNA methylation under nutrient (nitrate) stress in *P. tricornutum*, studies in green microalgae suggest that dramatic changes in DNA methylation tend to occur primarily during cellular division [43, 44]. In *C. cryptica*, cell growth ceases almost immediately after silicon starvation, while under N limitation, cell cycle progression will still proceed until full arrest occurs, and thus the immediate cessation of cell cycle under silicon deprivation may preclude changes in methylation.

Consistent with studies in other diatoms [41, 45], methylation is predominantly at CpG dinucleotides, with low levels of CHH and CHG methylation (Fig. 3a). By comparison, the nuclear genome of *T. pseudonana* is 2.57% methylated, and *Fragilariopsis cylindrus*, with a genome of 81 Mbp, is 8.63% methylated [45]. Based on this study and previous methylome studies in other microalgae, there appears to be no strict correlation between overall methylation and genome size, as well as no pattern of DNA methylation within an algal class; for example, the global per-cytosine methylation of the chlorophytes *Chlorella NC64A* and *Chlamydomonas reinhardtii* are 82.65 and <2% with genome sizes of 42 and 120 Mbp, respectively [44, 46, 47].

To assess what genomic features are methylated in *C. cryptica*, AUGUSTUS gene models and RepeatModeler data were overlaid against methylation levels across the largest assembled genomic contigs for both conditions. The majority of highly methylated regions aligned with repeat sequence (Fig. 3b; Additional file 1: Figure S5). This is consistent with other methylation studies which hypothesize that DNA methylation inhibits transposable element expansion and is commonly found across repeat regions [41, 48]. These highly repetitive/methylated regions were substantial, spanning as large as 30 kb (Fig. 3b; Additional file 1: Figure S5). Additionally, there were similarly sized hypomethylated regions that were gene-rich, and contained essentially no methylation. While the pattern of hypermethylated repetitive sequence and hypomethylated genic sequence is common, the striking large-scale binary distribution of genomic methylation has not been shown before in an algal species or other organisms and this unique genomic architecture may likely have influence on higher order chromatin structure in *C. cryptica*.

Methylation was minimal over gene sequences. There was a slight difference in the fraction of methylation in introns (3.23% on average) versus exons (4.14%) (Fig. 3c, d), a trend that is in agreement with that found in other organisms, including *P. tricornutum* [41]. Across the gene body, methylation gradually increased towards the 3′ direction and was lower 500 bp upstream compared to downstream (Additional file 1: Figure S6). Notably, there were higher levels of repetitive sequence in the 5′ upstream, hypomethylated region (Additional file 1: Figure S6), suggesting that these repeat elements are under a different control mechanism compared to the majority of repeats in the genome.

For genes with sufficient coverage to define a methylation status, 77% (averaged between both experimental conditions) were unmethylated and 23% were methylated (Additional file 1: Table S3). Methylated genes had an average methylation of 88% and had lower transcript levels with ~500 average FPKM across all transcriptomes. Unmethylated genes had an average methylation of 7% and had significantly higher levels of transcript (average ~4500 FPKM, Additional file 1: Table S3).

There was no linear correlation between fraction methylation and FPKM in either condition, however, three general populations appeared (Additional file 1: Figure S7). The first consisted of genes with very little transcript abundance (ranging from 0 to 10 FPKM), almost all of which (95%) were methylated (Additional file 1: Figure S7a, box i). The second population, similar to findings from [41], comprised the majority of genes which contained moderate to high transcript abundance and were largely unmethylated (Additional file 1: Figure S7b). Lastly, and somewhat surprisingly, there appeared to be several genes with moderate to high FPKM levels with high levels of methylation (average FPKM values ranging from 4600 to 161,000, Additional file 1: Figure S7b, box iii, Additional file 2). Functional annotation of these genes indicated enrichment in genes with transporter activity and/or an interaction with the cell surface. These included a nitrate transporter (g14234.t1), silicon transporter (g21035.t1), sodium-dicarboxylate symporter (g11380.t1, g10492.t1), sulfate transporter (g6964.t1), and a bacterially derived integrin (g18136.t1). In addition, genes involved in nitrogen metabolism, including the above nitrate transporter, as well as nitrate reductase (g22809.t1) and glutamate synthetase (g14060.t1) were also in this subpopulation. Highly methylated, highly expressed genes may be indicative of a unique role DNA methylation plays in these specific cellular functions. Methylation status of these genes did not change under our experimental conditions. Additionally, the absence of DNA methylation based silencing in these genes may indicate other epigenetic regulatory mechanisms that enable active transcription are at play. An example is histone tail acetylation, which has been shown to have a strong activating effect in *P. tricornutum* regardless of methylation status [49].

Investigations in *P. tricornutum* identified methylation shifts in nitrate metabolism genes under depletion conditions [41, 49], indicating that DNA methylation may play a regulatory role in nitrate metabolism in diatoms. We identified no significant change in methylation of genes pertaining to silicon metabolism or lipid accumulation under our conditions, instead there was a globally significant correlation of methylation between the two conditions. This data suggests that DNA methylation does not control the cell’s silicon status response in these conditions as is reported with nitrate metabolism in *P. tricornutum.* One explanation for these differences could be the fact that nitrogen stress on diatoms can cause severe cellular damage and detrimental effects, such as chlorosis and DNA and protein degradation [28, 50], whereas silicon starvation does not produce as severe phenotypes [32, 42, 51].

**Fig. 4:**
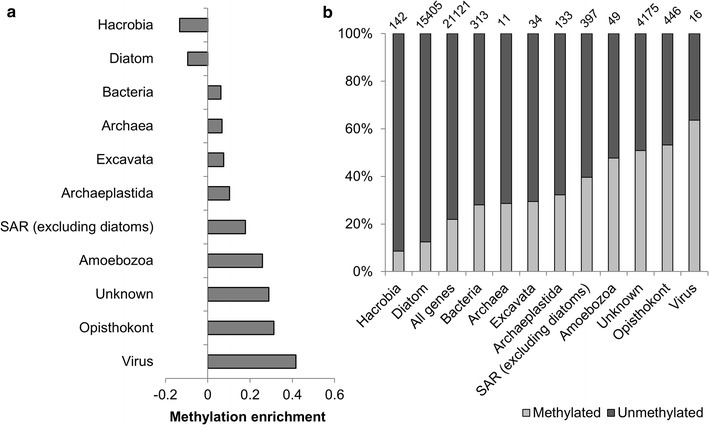
Methylation based on evolutionary origin of genes from DarkHorse analysis. **a** Enrichment analysis of methylated genes given their taxonomic group. Enrichment of methylation within a taxonomic group was determined by subtracting the proportion of genes methylated within a taxonomic group from the proportion of genes methylated across the *C. cryptica* genome. Groups with numbers below 0 have a lower proportion of methylated genes in the taxonomic group compared to the proportion of this group in the overall genome. **b** Percent of methylated and unmethylated genes relative to taxonomic group. *Top numbers* are the total number of genes from that group in the genome

We also determined whether there was a relation between the methylation status of genes and their evolutionary origin. We applied the DarkHorse analysis to identify possible horizontally transferred genes, and identified 1723 genes (41% of total methylated genes) that contained a top BLAST hit to diatoms, however, in comparing the proportion of diatom-derived genes within the subset of methylated genes, this number is significantly depleted relative to the proportion of diatom-shared genes in the whole genome (72% of the total, Fig. 4a). There was enrichment in methylated genes with no known taxonomic match (‘unknown,’ 1956 genes, 47% of total methylated genes versus 20% of all genes, Fig. 4b). There was also enrichment of methylated Opisthokont genes (Fungi, Metazoan, Choanoflagellida, Ichthyosporea), which were primarily annotated for transposon processes (Fig. 4a; Additional file 2). Within the methylated subset of genes, there was a slightly higher proportion of foreign genes (i.e., viral, bacterial) as well as genes ancestral to the cell, but not core to diatom function, indicated by loss in other diatom genomes (groups SAR excluding diatoms, Archaeplastida, Fig. 4b).

In general, methylated genes in *C. cryptica* are not highly expressed and appear to be non-essential under our conditions. Furthermore, there appears to be a slight preference to methylate genes that are horizontally acquired, perhaps as a silencing strategy to prevent potential detrimental effects of the foreign gene. Alternatively, it could also indicate that the mechanism for foreign gene insertion in eukaryotic microalgae, which is largely unknown, is more likely to occur in hypermethylated regions than in hypomethylated regions of the genome (Fig. 4b).

Lastly, using the same definitions of gene methylation as in Additional file 1: Additional methods, we compared methylation levels of genes in *T. pseudonana* from [5, 45] to the 0 h methylome of *C. cryptica*. Only 139 genes (1% of the total) in *T. pseudonana* were methylated and out of 24 *T. pseudonana* methylated genes with orthologs in *C. cryptica*, only five were methylated in both diatoms. Shared methylated genes were annotated as reverse transcriptases (g23181.t1, g15433.t1), and transposase IS4 (g9451.t1). The significantly low number of genes with shared methylation status in the two diatoms suggests a conserved role for methylation in these centric diatoms to mask and silence mobile DNA elements. Furthermore, while DNA methylation does not appear to temporally regulate genes in *C. cryptica* under our conditions, the comparison of these two diatom methylomes showed that both were associated with transposable element silencing. It has been demonstrated in higher plants that transposable element silencing has effects on the expression of nearby genes [52]. The methylation of the few genes found in hypermethylated regions (Fig. 3b) in *C. cryptica* may have resulted from the methylation of a nearby transposable element insertion and any resulting changes in gene expression could have downstream effects on the metabolism and function of that organism.

### Predicted subcellular localization of enzymes

**Fig. 5:**
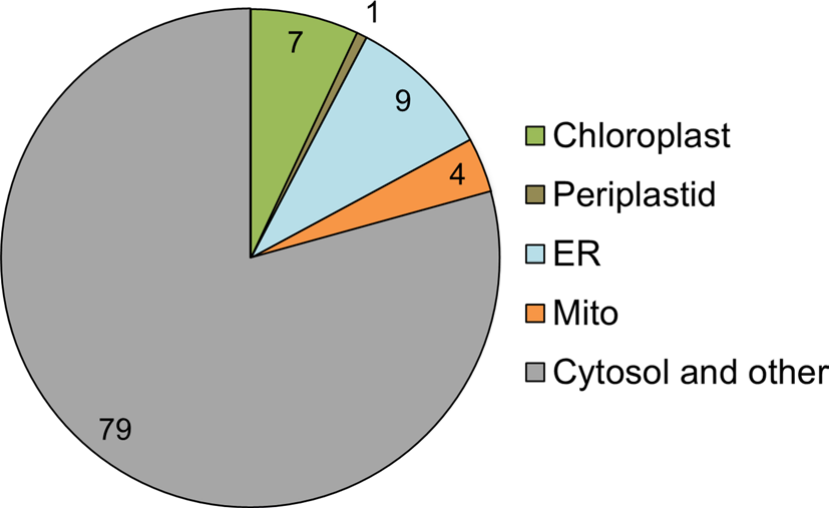
In silico targeting predictions of all nuclear gene models in *C. cryptica.* Percentages are listed in the chart. See also Additional file 2, subcellular targeting. targeting predictions used for this specific analysis were SignalP 3.0, TargetP, ChloroP, HECTAR, Predotar, and ASAFind [43, 44, 55–58]

An overarching theme in the study of diatom metabolism is the diversity of intracellular localization of enzymes in different organelles: the mitochondrion, cytosol, chloroplast, ER, and the periplastid compartment [22–24, 26, 27, 53]. The latter of these compartments is unique to photosynthetic eukaryotes that have undergone a secondary (or greater) endosymbiotic event. In addition, specific to heterokonts, the ER is extended and surrounds the chloroplast. Using in silico tools as a means to map the location of metabolic processes in the cell provides a clearer description of metabolic function and transport of metabolites and proteins in and out of these organelles and information for accurate modeling. We used SignalP 3.0, TargetP, Predotar, ChloroP, HECTAR, and ASAFind to predict which proteins are putatively targeted to the organelles in *C. cryptica* (Fig. 5; Additional file 1: Additional methods; [54–58]). The first four in silico organellar prediction programs are developed for higher plants and other eukaryotes, but we have found their collective use to be suitable for some basic understanding of metabolic compartmentation in diatoms, particularly when overlaid with coordinate expression patterns of particular genes [27, 32]. The latter two programs, HECTAR and ASAFind are specific for heterokonts, and take advantage of the conserved amino acid sequence motif (ASA-FAP) which is part of the N-terminal bipartite signal peptide that is unique to these genera of algae. Given sequence manipulations [22–24] that have identified specific amino acid substitutions within that conserved motif resulting in altered organellar targeting of an enzyme, it is feasible to use these prediction programs to hypothesize which proteins are partitioned into the chloroplast versus periplastid compartment. Because of the uniqueness of the periplastid compartment, we focused our analysis on proteins targeted there. Targeting of enzymes to other compartments will be described in the context of the metabolic processes analysis below. We identified a total of 120 proteins with targeted prediction to the periplastid compartment (Additional file 3).

Moog and colleagues [24] characterized the predicted proteome of the periplastid compartment (PPC) in *P. tricornutum* and concluded that some important cellular functions occurred there, specifically with regard to transport and protein import processes into the chloroplast, but that very few protein components of housekeeping biochemical pathways were present. Using direct localization approaches, they also documented that only 55% of proteins predicted to be targeted to the PPC actually were confirmed to be there, highlighting the poor state of predictive programs, perhaps due to transmembrane domains near the N-terminus, or mis-predicted signal cleavage sites.

Our analysis generally corroborated the findings of [24], with some exceptions, and potential new findings (Additional file 3). We identified one protein (g12899.t1) with an ANTH domain which may be putatively involved in clathrin assembly, as well as a dynamin, which is likely involved in chloroplast division (g1668.t1), suggesting that components of clathrin-mediated vesicular trafficking may be present in the PPC. Another protein, g23203.t1, is a member of the S2P/M50 family of regulated intramembrane proteolysis proteases, which use proteolytic activity within the membrane to transfer information across as a means to integrate gene expression with physiologic stresses occurring in another cellular compartment [59]. Mechanisms of cross-talk between the cytoplasm and chloroplast of diatoms have not been well addressed in the literature, but of necessity, would require the involvement of the PPC.

Lastly, phosphoenolpyruvate carboxylase (PEPC—g7839.t1) and several carbonic anhydrases were predicted to be PPC targeted, consistent with a mechanism to recapture CO_2_ lost from the chloroplast by inefficient carbon fixation assimilation and incorporate it into oxaloacetate. Experimental validation using genetic constructs to confirm localization of predicted enzymes in the PPC and other organelles is needed to confirm in silico analyses. Nevertheless, the prediction programs, particularly on a proteome with accurate prediction of correct N-termini based on RNAseq data, offer a starting point to further characterize the significance of the PPC in diatoms.

### Comparative analysis of glycolysis, gluconeogenesis, and the pyruvate hub

Glycolysis, the catabolism of glucose to produce pyruvate, and the reverse pathway gluconeogenesis, are core metabolic pathways which process photosynthetically fixed carbon into compounds and energy for use by the cell. Pyruvate metabolism is involved in the distribution of carbon to different cellular processes, which include biosynthesis of compounds and energy generation. While genes and predicted subcellular localization of steps in these processes were generally conserved between the oleaginous *C. cryptica* and relatively non-oleaginous *T. psuedonana,* there were several important differences in the architecture of the pyruvate hub in three different intracellular locations.

**Fig. 6:**
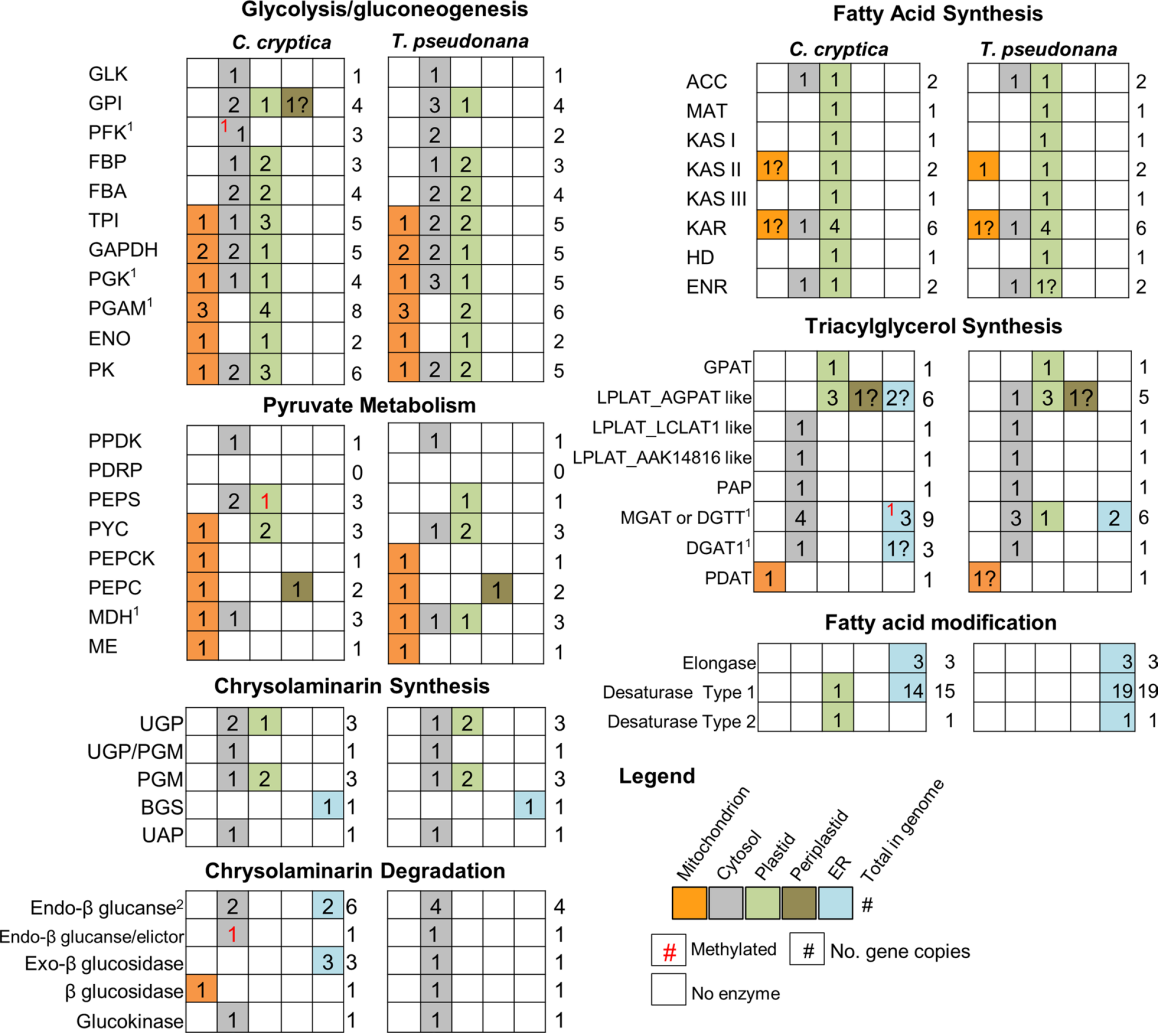
Comparative analysis of key carbon metabolic pathways between *C. cryptica* and *T. pseudonana*. *Different colored boxes* denote subcellular location of a given enzyme based on bioinformatic targeting predictions. *Number* within the *box* indicates how many enzyme copies are targeted to that location. *Numbers* to the *right of boxes* are total gene copy number found in the genome. *Question marks* indicate weak targeting and/or inconsistencies between targeting prediction programs. *Superscript numbers* on enzymes indicate the number of genes found with partial sequence in *C. cryptica* at the N-terminus, thereby not containing targeting prediction. *Red numbers* are methylated genes. In the case of MGAT or DGTT, one of the four ER predicted genes is methylated. *AAK14816-like* putative glycerol-3-phosphate acyltransferase, *ACC* acetyl-CoA carboxylase, *AGPAT* 1-acyl-glycerol-3-phosphate acyltransferase, *BGS* 1,3 β-glucan synthase, *DGAT* diacylglycerol acyltransferase, *DGTT* diacylglycerol acyltransferase type 2, *ENO* enolase, *ENR* enoyl-ACP reductase, *FBA* fructose bisphosphate aldolase, *FBP* fructose 1,6 bisphosphatase, *GAPDH* glyceraldehyde 3-phosphate dehydrogenase, *GLK* glucokinase, *GPI* glucose-6-phosphate isomerase, *GPAT* glycerol-3-phosphate acyltransferase, *HD* 3-hydroxyacyl-ACP dehydratase, *KAR* 3-ketoacyl-ACP reductase, *KAS* 3-ketoacyl-ACP synthase, *LCLAT1* lysocardiolipin acyltransferase 1, *LPLAT* lysophospholipid acyltransferase, *MAT* malonyl-CoA-ACP transacylase, *MDH* malate dehydrogenase, *ME* malic enzyme, *MGAT* monoacylglycerol acyltransferase, *PAP* phosphatidic acid phosphatase, *PYC* pyruvate carboxylase, *PDRP* pyruvate phosphate dikinase regulatory protein, *PEPC* phosphoenolpyruvate carboxylase, *PEPCK* phosphoenolpyruvate carboxykinase, *PEPS* phosphoenolpyruvate synthase, *PFK* phosphofructokinase, *PGAM* phosphoglycerate mutase, *PGK* phosphoglycerate kinase, *PGM* phosphoglucomutase, *PK* pyruvate kinase, *PPDK* pyruvate phosphate dikinase, *TPI* triose phosphate isomerase, *UAP* UDP-*N*-acetylglucosamine pyrophosphorylase, *UGP* UTP-glucose-1-phosphate uridylyltransferase

**Fig. 7:**
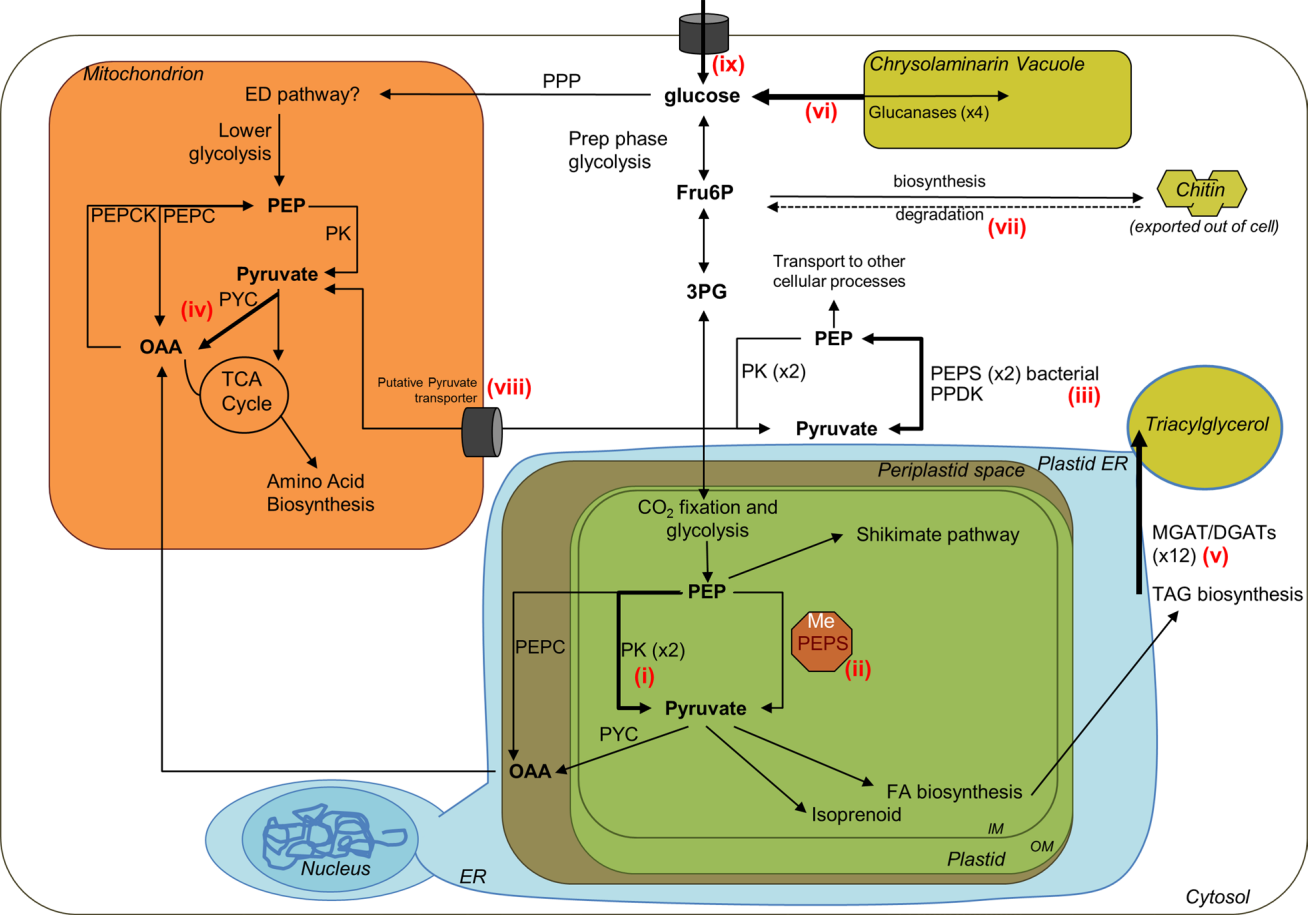
Metabolic overview highlighting key findings from *Cyclotella cryptica* genome. *Red numbers* are referred to in the text. Enzymatic abbreviations are listed in Fig. 5. *ED* Entner–Doudoroff, *IM* inner membrane, *OM *outer membrane, *PEP* phosphoenolpyruvate, *Fru6P* fructose-6-phosphate, *3PG* glycerate 3-phosphate, *OAA* oxaloacetate, *PPP* pentose phosphate pathway

*Cyclotella cryptica* has an additional copy of plastid-localized pyruvate kinase (Figs. 6, 7i), a key glycolytic regulatory enzyme that catalyzes the unidirectional conversion of phosphoenolpyruvate (PEP) and ADP to pyruvate and ATP. In addition, the gene encoding plastid-localized PEP synthase (PEPS) which catalyzes the reciprocal reaction to produce PEP from pyruvate, is heavily methylated (96% fraction methylation) with low transcript (average FPKM 0.61) abundance. This suggests that under the conditions tested, PEPS may be silenced by methylation (Fig. 7ii), and can be assumed to be non-functional. In contrast, the plastid PEPS gene is homologous to the single copy of PEPS found in *T. pseudonana*, which shows moderate levels of expression and is unmethylated (0.19% across gene body) under similar experimental conditions [32, 45].

In *C. cryptica,* there are two isozymes of PEPS localized in the cytosol not found in *T. pseudonana* (Figs. 6, 7iii). Generation of PEP from pyruvate in the cytosol instead of the plastid would separate a reaction that utilizes pyruvate from a reaction that produces pyruvate, potentially improving the efficiency of pyruvate utilization for processes such as fatty acid synthesis in the plastid of *C. cryptica*. The methylation of PEPS, along with additional copies of PEPS in the cytosol might further allow for an increased degree of fine-scale regulation of this important node of metabolism. The extra copy of plastid-localized PK in *C. cryptica* relative to *T. pseudonana* could indicate a greater potential for pyruvate production in *C. cryptica*, possibly translating into a greater fatty acid synthesis capability (Fig. 7).

There were also differences in the mitochondrial pyruvate hub between the two species. *T. pseudonana* lacks a mitochrondrial-localized pyruvate carboxylase (PYC), which converts pyruvate to oxaloacetate (OAA), yet possesses a cytosolic copy, whereas *C. cryptica* has a mitochondrial but not cytoplasmic PYC (Figs. 6, 7iv). This suggests that *C. cryptica* can produce OAA via pyruvate in the mitochondria, but *T. pseudonana* would have to generate it elsewhere then import into mitochondria, via a malate–aspartate shuttle. In addition to the differential requirement for transport, metabolic drivers for possible mitochondrial gluconeogenesis may differ in the two species, since in *C. cryptica,* gluconeogenesis could be initiated within the mitochondrion by conversion of pyruvate to PEP by the combined action of PYC and PEPCK.

### Lipid metabolism

The genetic basis for enhanced lipid productivity in *C. cryptica* was investigated by identifying genes for fatty acid and TAG biosynthesis, as well as fatty acid modification biosynthesis, and comparing this repertoire with that found in *T. pseudonana.* An identical suite of plastid-localized essential fatty acid biosynthesis orthologs from *T. pseudonana* were found in *C. cryptica* (Fig. 6). There were differences in the TAG biosynthesis enzyme inventory between the two diatoms, such as the presence of several additional genes found in *C. cryptica* and subcellular localization predictions (Fig. 6). An additional three genes possessing an LPLAT_MGAT-like domain found in either MGAT or DGTT enzymes were present in *C. cryptica*. One of these putative MGAT/DGTT (g3706.t1) had highly abundant transcripts (average FPKM of 23,833). Another putative MGAT/DGTT (g21947.t1) is a homolog to the *P. tricornutum* DGTT2b, which has been tested as a target for genetic manipulation to increase TAG content [60, 61]. There were also two additional copies of DGAT1: one with predicted targeting to the ER (g23184.t1), and the other (g9565.t1) was a partial sequence, hence targeting could not be determined. The additional copies of these enzymes in *C. cryptica* may indicate an increased ability to generate TAG and/or allow for finer control over TAG production in the case that these enzymes differ in their specificity, regulation or kinetic properties (Fig. 7v).

Omega-3 long chain polyunsaturated fatty acids (LC-PUFAs) are a high-value product that is of interest for nutritional purposes [5]. *Cyclotella cryptica* synthesizes abundant LC-PUFAs including over 16% eicosapentaenoic acid (EPA) and 4% docosahexaenoic acid (DHA) of the total fatty acids; generally a higher level than in other algal species [5]. We investigated if the higher capacity to produce these biotechnologically relevant fatty acids was due to an increase gene number within the fatty acid elongases and desaturases, which encode for the enzymes that catalyze the biosynthesis of LC-PUFAs. Overall, elongases and desaturases potentially involved in LC-PUFA synthesis were highly conserved between *C. cryptica* and *T. pseudonana* (Fig. 6). Although *C. cryptica* contained fewer copies of type 1 desaturases, it had all the types including Δ5, Δ6, Δ8, Δ9, Δ11 and Δ12. Both diatoms contain a single copy of a Δ4 desaturase that catalyzes the addition of the sixth double bond to docosapentaenoic acid (22:5) chain to make DHA (C22:6) [20]. The existence of the same types of elongases and desaturases in both diatoms suggest that the greater ability of *C. cryptica* to produce EPA compared to *T. pseudonana* is not due to an increase in isozymes in the genome and could be more dependent on the flux of carbon from upstream processes into this pathway.

### Carbohydrate biosynthesis and degradation

Chrysolaminarin, the main form of storage carbohydrate in diatoms, is a water soluble β-(1-3) linked glucan, and is stored outside of the plastid in a large vacuole [36, 62]. In diatoms, genes involved in biosynthesis and degradation of chrysolaminarin have been annotated, and the biochemistry of key enzymatic steps has been characterized [10, 53, 63]. The gene repertoire for chrysolaminarin biosynthesis enzymes is highly conserved between *T. pseudonana* and *C. cryptica* in terms of sequence similarity and predicted targeting (Fig. 6). Like other stramenopiles, *C. cryptica* possesses a fused cytosolic phosphoglucomutase/UTP-glucose-1-phosphate uridyltransferase, which carries out the first two-enzymatic steps toward chrysolaminarin production [53]. Both *T. pseudonana* and *C. cryptica* also possess three individual copies of phosphoglucomutase and UTP glucose-1-phosphate uridyltransferase similarly distributed between the plastid and cytosol. The metabolic significance of the fused gene and multiple single gene copies leading to chrysolaminarin production is unknown, yet might provide metabolic flexibility by creating several routes toward synthesis that differ in organellar location (plastid vs. cytosol), carbon transporter specificity out of the plastid (Glu-6P versus UDP-glucose), and enhanced efficiency or substrate channeling (fused protein versus single reaction proteins). While these enzymatic steps have an intriguing metabolic flexibility, the final step in chrysolaminarin production, catalyzed by a beta-glucan synthase, does not. The β-glucan synthase is present as a single copy and highly conserved in all sequenced diatom genomes.

In contrast to the chrysolaminarin biosynthetic pathway, genes coding for enzymes involved in the degradation of chrysolaminarin were poorly conserved between *T. pseudonana* and *C. cryptica* with an average low percent identity between homologs (51%). The *C. cryptica* genome encodes four additional enzymes putatively involved in chrysolaminarin breakdown compared to *T. pseudonana* (two additional endoglucanases, two additional exoglucosidases, Figs. 6, 7vi). Of the three ER predicted exoglucosidases, two were found only in *C. cryptica,* presumably recently acquired by horizontal transfer. The most highly expressed exoglucosidase in *C. cryptica* (g19489.t1) is unique, with low similarity compared with those of higher plants and fungi. In addition to the two-glycosyl hydrolase domains responsible for carbohydrate breakdown, the gene also contains two fascin-like domains, putatively involved in actin crosslinking, which implies a positioning component to its function. The increased number of chrysolaminarin degradation enzymes in *C. cryptica* compared to *T. pseudonana* may indicate a greater capacity to break down β-1,3 glucans, allowing for more carbon substrate to be used for the production of triacylglycerol or other carbon products.

### Chitin biosynthesis and degradation

**Fig. 8:**
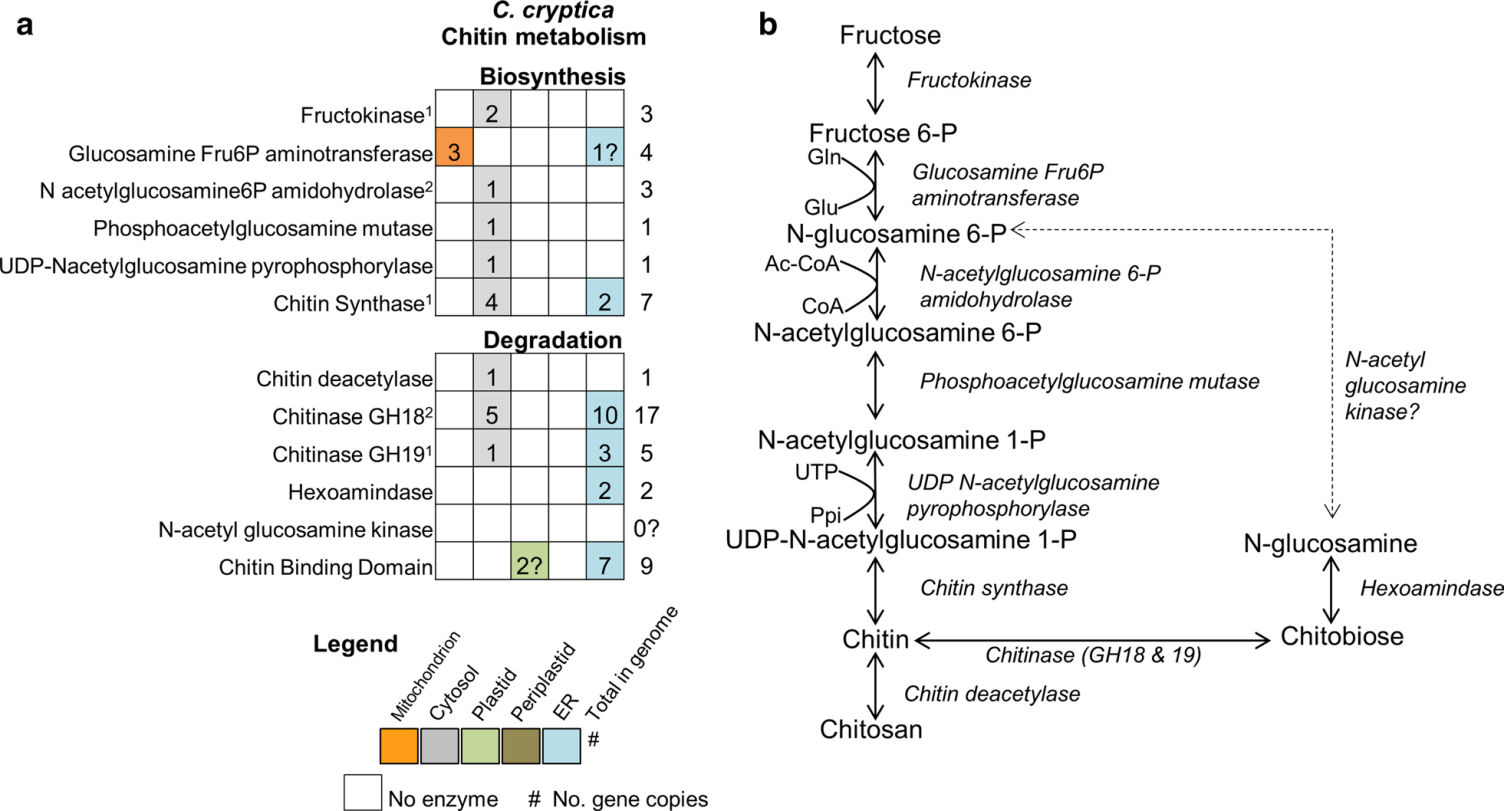
Chitin metabolism in *C. cryptica.* **a** Genes and their predicted subcellular localization. Organizational schematic as described in Fig. 6. **b** Hypothetical chitin biosynthesis and degradation pathway in *C. cryptica. Dashed line* indicates that the enzyme is not found in *C. cryptica*

Chitin, or poly β-(1,4) linked *N*-acetylglucosamine, is the most abundant polymer in the ocean, and second most on the planet [64, 65]. The exoskeleton of arthropods and insects is made of chitin, and chitin is also produced by many diatoms where it has been proposed to control buoyancy and/or be involved in silica cell wall synthesis [66–68]. Chitin is estimated to comprise over 30% of the mass of the cell in some diatom species [69], which is a significant reservoir for cellular carbon that could be shunted into other pathways to facilitate productivity in a controlled cultivation environment. However, little is known about the cellular role of chitin, the biosynthetic pathway is poorly characterized, and annotation of relevant genes in diatoms is incomplete. In *C. cryptica*, most enzymes involved in synthesis and degradation of chitin are predicted to be cytosolic or ER targeted (Fig. 8)—the latter may indicate extracellular secretion, plasma membrane localization, or an association with the silica deposition vesicle.

Chitin fibrils are apparently located between the silica frustule and plasma membrane [69], yet the intracellular site of chitin production is unknown, therefore we investigated the targeting of enzymes predicted to be involved in this process for additional insight. The first and rate-limiting enzyme of the chitin synthase complex is glucosamine fructose 6-P aminotransferase, of which *C. cryptica* contains four putative copies (Fig. 8a). Three of those copies are predicted by several programs to have mitochondrial targeting, perhaps suggesting direct utilization of mitochondrial-produced glutamine from the glutamate–glutamine cycle. However, fructose-6P is not produced in the mitochondria and would need to be transported there. *Cyclotella cryptica* GFATs all also have less well supported ER signal peptides, which may also indicate targeting through the ER to a different cellular location. In both *T. pseudonana* and *C. cryptica* there are three steps in the pathway represented by a single isozyme, all with cytosolic targeting prediction. The final step of chitin biosynthesis polymerizes the *N*-acetyl-d-glucosamine molecules by chitin synthase (CS) to produce chitin. There are five full copies of CS, which contain the chitin_synth_C domain, and two partial sequences in *C. cryptica* (Fig. 8a). These Division 2 chitin synthases have been found only within *Thalassiosirales* diatoms, of which *C. cryptica* is a member, and were distinct from the Division 1 enzymes found in the pennate diatoms [70, 71]. Since *Thalassiosirales* all secrete chitin fibrils, whereas pennate species do not; these chitin synthases could be involved with fibril formation.

*Cyclotella cryptica* has a nearly complete chitin degradation pathway including a substantial number of chitinases, 22 in total, which perform the first step in chitin degradation (Fig. 8). Similar numbers were reported in *T. pseudonana* [68]. Most of the chitinases are shared within the *Thalassiosirales* group and have similarity to either bacterial (13 copies) or fungal (7 copies) chitinases. It is unknown when or why diatoms would break down chitin, but the number of chitinase genes combined with their general transcript abundance in *C. cryptica*, suggests that they play an important if not as yet elucidated role in cellular processes. Four chitinases also contain one or two peritrophin A domains. In insects and other multicellular organisms, peritrophin is a protein embedded within a chitinous membrane (the peritrophin matrix) which separates digested food from the midgut epithelium where it is hypothesized to aid in digestion, protect against pathogens, and provide mechanical support [72]. In *T. weissflogii*, freeze fracture images of the space between the plasma membrane and silica frustule revealed structures with the appearance of chitin fibrils [69], which raises the possibility that peritrophin-containing chitinases in *C. cryptica* may be found in a similar matrix as an organic component of the cell wall. This might suggest that a dynamic processing of chitin, involving synthesis and degradation, occurs in this location. In addition to the chitinases, other chitin binding genes in *C. cryptica* possessed Peritrophin A domains which have also been identified in *T. pseudonana* [73].

Two recently duplicated gene copies encoding the subsequent step in chitin degradation, the breakdown of chitobiose catalyzed by hexosaminidase, are ER targeted in *C. cryptica* (g14774.t1, g22243.t1). A homolog could not be identified in *T. pseudonana*. Furthermore, even with a manual search, we were unable to identify a kinase that phosphorylates the acetyl glucosamine molecule to break down chitin to fructose-6-phosphate in any diatom genome (Fig. 8). It is possible that *N*-acetylglucosamine kinase was not well annotated and/or is distinct from characterized copies, making it difficult to identify, but may also suggest that diatoms lack the ability to breakdown chitin to a form that can be fed into glycolysis/gluconeogenesis, or possess a novel mechanism to do so (Fig. 7vii).

### Carbon transport within the cell

Intracellular carbon flux relies on the transport of metabolites between organelles, which connects otherwise disjointed pathways. An annotation of carbon transporters is essential to develop a more accurate metabolic map of carbon flux. This may be especially important in diatoms, which are highly compartmentalized as a result of their origin through secondary endosymbiosis. The *C. cryptica* genome was screened to identify a provisional list of intracellular organic carbon flux transporters by selecting Pfam and Reactome annotations containing matches to any mono- or disaccharide molecule. Examples of annotated transporters included the Major Facilitator Superfamily (MFS), Sugar and other transporter (MFS, Pfam00083), Triose-phosphate/phosphate translocator (TPT, Pfam03151), or the UAA transporter family (Pfam08449). TPT and UAA are specific for monosaccharide-based molecules, with the latter also including transporters for UDP-*N*-acetylglucosamine, a component of chitin. The MFS transporters have diverse substrate specificity, but the multi-domain Pfam00083 can include simple sugar phosphates. Homologs were identified for nearly all putative TPTs identified in *P. tricornutum,* (TPT1-TPT13) ([74]; Additional file 4). Using the same naming conventions as [74], *C. cryptica* has an additional TPT2, TPT4b (named TPT4c, g4485.t1), and TPT8 (no PFam domain, but high similarity TPT8), as well as three additional genes containing the TPT domain, of which two contain homologs in *T. pseudonana*, and one is unique to *C. cryptica* −g10651.t1. Neither of the TPT2 genes in *C. cryptica* has a bipartite leader sequence, whereas the *P. tricornutum* proteins were directly localized to the periplastid/cER membrane [74]. While it is possible that either or both TPT2 in *C. cryptica* are localized to the periplastid/cER membrane, the potential differential targeting between the two diatoms could imply an alteration of carbon flux processes. In addition, the presence of another chloroplast targeted TPT4b, could indicate differences in flux into and out of the plastid or differential regulation [74] compared to *P. tricornutum* (Additional file 4).

Given the possible differences in pyruvate processing between *C. cryptica* and *T. pseudonana* described earlier, we investigated the complement of pyruvate transporters in both species. We identified an ortholog to the sodium coupled pyruvate transporter identified in *Arabidopsis* and subsequently in *T. pseudonana* which is proposed to transport pyruvate into the plastid envelope (g5998.t1) [63, 75]. We also identified a putative mitochondrial pyruvate carrier (MPC), which is orthologous to the MPC1 and 2 identified in humans, *Drosophila*, and yeast [76, 77] the first of its kind identified in diatoms. While in other organisms the complex is formed of two or three subunits, it appears that *C. cryptica* and other diatoms have a fused single gene that contains both MPC 1 and 2 (g13004.t1). MPC is thought to be a significant source of pyruvate transport into the mitochondria to be fed into the TCA cycle. However, since diatoms should be able to generate pyruvate via mitochondrial glycolysis (Fig. 6, [27, 53]), the role of this pyruvate transporter may be different, perhaps in diatoms it can transport pyruvate out of the mitochondria (Fig. 7viii).

**Fig. 9:**
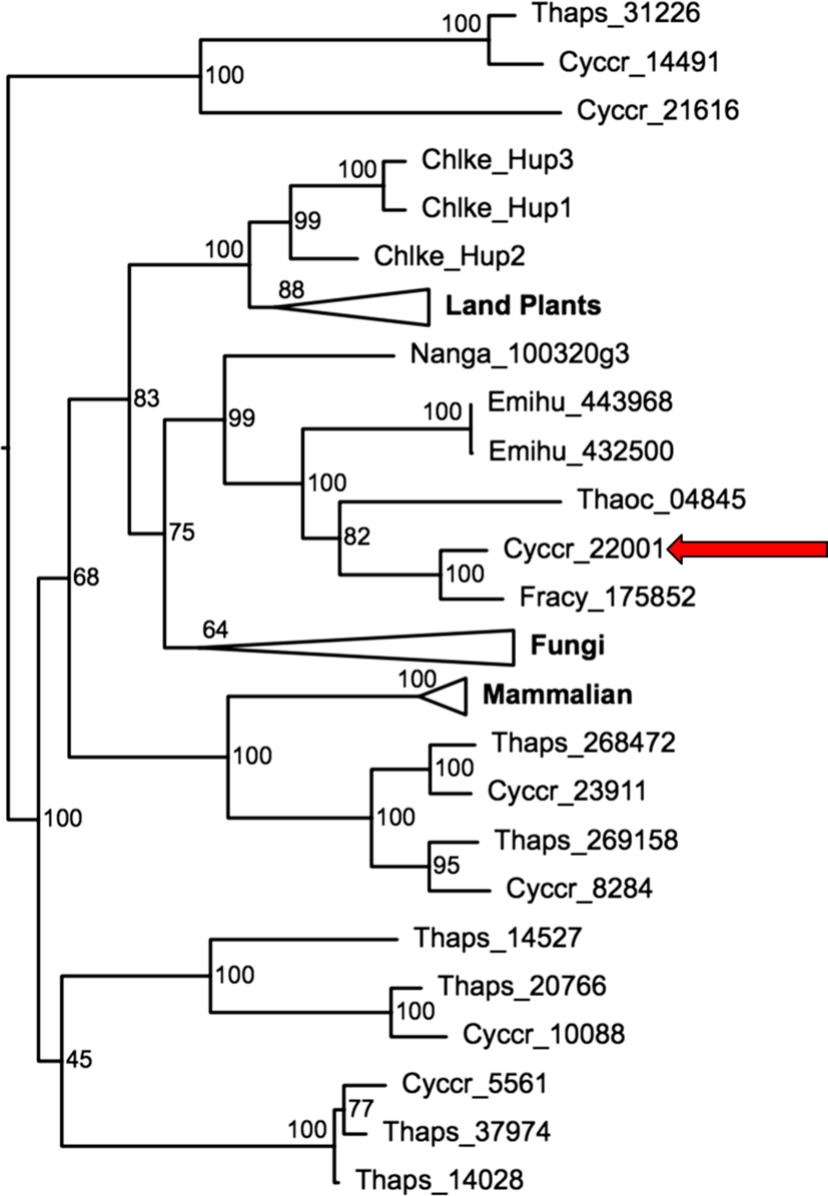
Phylogenetic analysis of hexose transporters in *C. cryptica (Cyccr)* and *T. pseudonana (Thaps)*. All genes contain PF00083 Sugar (and other) transporter domain. Highlighted gene is hypothetical heterotrophic glucose transporter in *C. cryptica* and with nearest matches to that transporter found in the diatoms *T. oceanica, F. cylindrus,* the haptophyte *E. huxleyi,* and stramenopile *N. gaditana.* Phylogeny was generated using default parameters in RAxML_GUI v1.3 and is mid-point rooted. Abbreviations and accession numbers are found in Additional file 4

A distinguishing metabolic feature of *C. cryptica* is the ability to grow truly heterotrophically on d-glucose and d-galactose, while other diatoms with sequenced genomes are thought to be obligate autotrophs [78–81]. Heterotrophic growth is useful for maximizing growth rates and attainable biomass in large scale production; by reducing respiratory losses at night or a strict dependence on light regimes [82]. Active transport of glucose or carbon substrates at the plasma membrane via the activity of hexose transporters is required for heterotrophic growth. This was demonstrated by the trophic conversion of *P. tricornutum* from obligate phototrophy to facultative heterotrophy through introduction of a hexose transporter [83]. Seven candidate genes in the *C. cryptica* genome encoded putative triose or hexose transporters [84] (Fig. 9). Orthologs of most of these were found in the obligate phototroph *T. pseudonana*, suggesting that they are likely involved in intracellular sugar transport. A *T. pseudonana* ortholog could not be identified for the most highly expressed putative sugar transporter (g22001.t1). The top BLASTp hits to this predicted sugar transporter were to characterized hexose transporters in yeast, and this gene clustered with hexose transporters in *Chlorella kessleri* (abbreviated Chkle), higher plants, and yeast (Fig. 9). There is low similarity (37% identity) to a partial gene in the diatoms *T. oceanica* (Thaoc_04845) and *Fragilariopsis cylindrus* (Fracy_175852), as well as hits to other microalgae *E. huxleyi* (Emihu) and *N. gaditana* (Nanga), with similar percent identities. We hypothesize that this gene is a good candidate for encoding a transporter responsible for glucose uptake in *C. cryptica* (Fig. 7ix).

### Tool development for genetic manipulation and microscopy

**Fig. 10:**
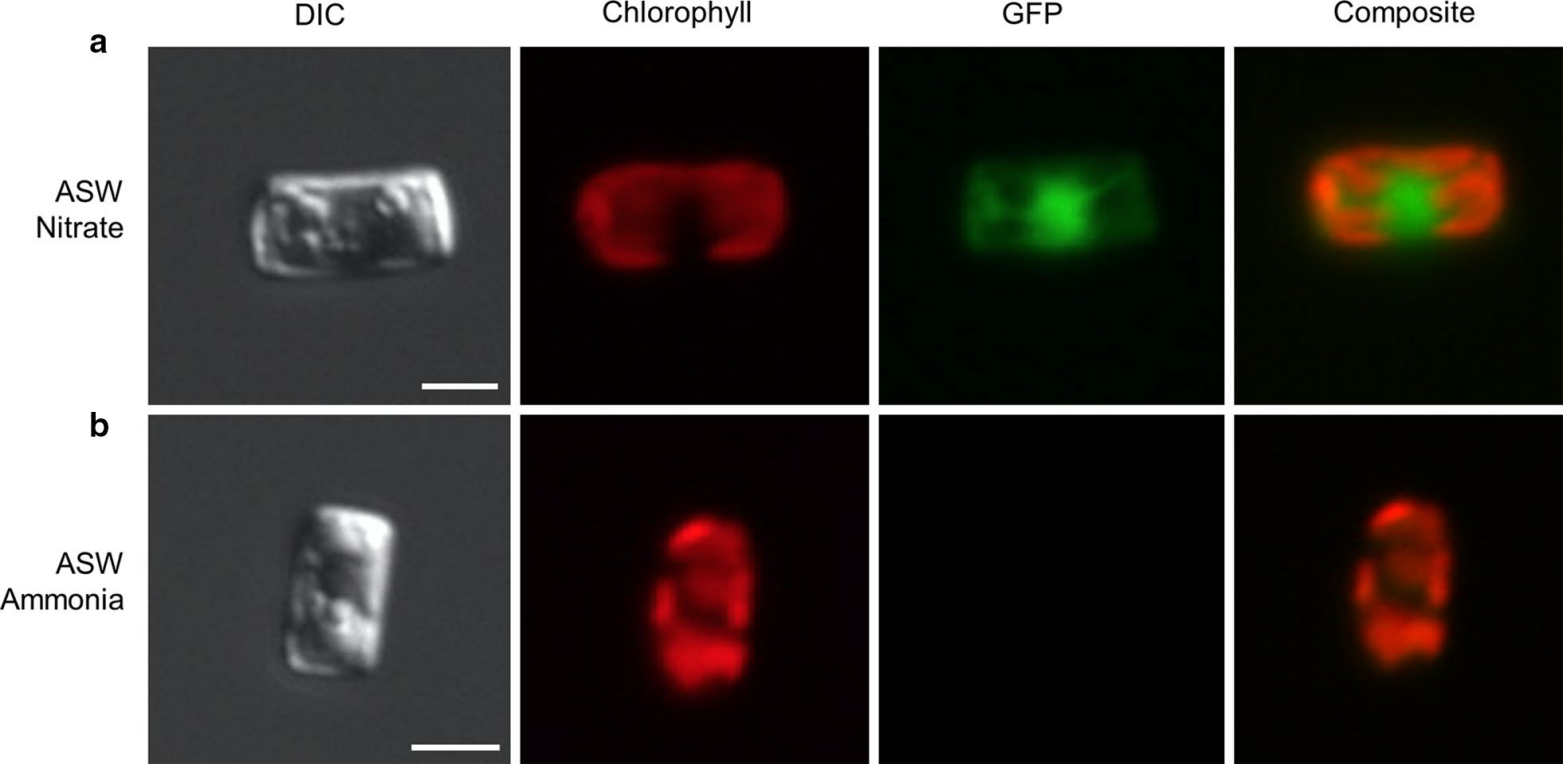
Genetic manipulation in *C. cryptica.* Conditional expression using the nitrate reductase promoter. **a** GFP expression in ASW media, with nitrate as the nitrogen source. **b** No GFP expression in repressive modified ASW media, with ammonia as the nitrogen source. Chlorophyll and GFP are falsely colored *red* and *green*, respectively. Composite images are 3D reconstructions. *Scale bars* 5 μm

The use of genetic engineering tools is crucial for the development of industrially relevant species for production because it allows investigation of cellular metabolism and enables enhancement of the species’ characteristics to increase production efficiency. While successful nuclear transformation of *C. cryptica* was achieved during the ASP [12], there has been little molecular work since. To improve the tools available for *C. cryptica,* we further adapted the nuclear transformation procedure, tested two *T. pseudonana* expression vectors and constructed one using *C. cryptica* sequences (Additional file 1: Additional methods). Two vectors allow for constitutively high expression, one of which uses native *C. cryptica* ribosomal protein L41 promoter and terminator sequences, while the second vector was derived from the *T. pseudonana fcp* gene [13]. The third vector, utilizing the *T. pseudonana* nitrate reductase (NR) promoter and terminator enables conditional expression, being induced upon transfer of nitrogen source from ammonia to nitrate [85]. Imaging of GFP under control of the NR promoter in *C. cryptica* grown in nitrate and ammonium is shown in Fig. 10. The larger size of *C. cryptica* (8–10 μm) compared to *T. pseudonana* (4–5 μm) and *P. tricornutum* (4–5 μm) may facilitate microscopy for identification of subcellular localization of enzymes. While vectors with native promoters and native sequence are important in some contexts, the successful utilization of pre-existing molecular tools from *T. pseudonana* in *C. cryptica* reduces effort and could facilitate development of specific applications.

## Conclusions

Determination of the genome sequence of *C. cryptica* elucidates metabolic features that underlie its high productivity and lipid accumulation ability, and the application of state-of-the-art approaches to further develop this species featured in the Aquatic Species Program for biofuel/bioproduct production [4]. Diatoms in general have attractive characteristics for large-scale biofuel/bioproduct production. In addition to their innate high productivity, their distinctive lipid induction stimulus by silicon starvation leads to rapid and substantial increases in triacylglycerol levels [6, 26, 42] without detrimental effects on cellular protein content that would typically occur under nitrogen starvation conditions used in other microalgae. Thus, they could serve as both a biofuel precursor and protein source in a production system.

A dominant feature of the *C. cryptica* genome is the presence of large regions of repeat sequences encoding a variety of types of mobile genetic elements (Additional file 1: Table S2) which are highly methylated, presumably as a means to inhibit expansion of these elements. In a global sense, in the conditions tested, methylation does not appear to play a significant role in regulating metabolic gene expression, however, there are notable and important exceptions in the examples of highly methylated yet highly expressed genes (Additional file 1: Figure S7), and the chloroplast-localized PEPS gene which is highly methylated and not expressed (Fig. 7ii). There are a few, yet significant, differences in gene content and metabolic topology when comparing *C. cryptica* and *T. pseudonana*, which may underlie the former’s high productivity. In particular, pyruvate metabolism is substantially different, with *C. cryptica* potentially being able to process pyruvate with greater efficiency because of compartmental separation of distinct reaction steps comparing the chloroplast and cytoplasm, and intercompartmental processing in the mitochondria, avoiding a transport step (Figs. 6, 7). Additional isozymes involved in TAG synthesis and carbohydrate breakdown are present in *C. cryptica*, which may improve carbon flux into TAG synthesis (Figs. 6, 7v, vi). Characterization of these metabolic differences, and those involved in other potential carbon sinks such as chitin, identifies a variety of gene targets to manipulate to directly test these hypotheses, and to understand how the differences in carbon flux may affect productivity, with a goal of ultimately improving productivity. The heterotrophic capability of *C. cryptica* may further aid in increasing productivity, either by enabling heterotrophic production or reducing respiratory losses at night from photosynthetically-fixed carbon. The application of state-of-the-art genetic tools to *C. cryptica* (Fig. 10), and continued development of such, will enable reprogramming of the metabolism otherwise adapted for environmental survival to improve productivity in the relatively controlled conditions for large-scale production. By bringing the promising productivity characteristics of *C. cryptica* in line with current day approaches to improve productivity, its potential as a model biofuel/bioproduct organism can be maximized.

## Methods

### Source of genetic material

Genomic DNA for genome and methylome sequencing was isolated from a clonal culture of *C. cryptica,* CCMP332. This strain of *C. cryptica* was originally isolated in June of 1956 from the West Tisbury Great Pond (41.355° N, 70.655° W) in MA, USA and maintained and by the National Center for Marine Algae and Microbiota (NCMA, formerly Provasoli–Guillard National Centre for Culture of Marine Phytoplankton CCMP). CCMP332 has been maintained in the lab since 2008 under a 12:12 light–dark cycle at 18 °C with an illumination of 150 μmol/m^2^ s light. For genomic DNA isolation, *C. cryptica* was plated onto artificial seawater (ASW) agar plates [86] under the presence of 1:1000 dilution of penicillin/streptomycin (Gibco, catalog no. 15140-122) to minimize bacterial contamination. A single colony was picked and grown in liquid ASW for scale up. 1 L liquid cultures were mixed using a magnetic stir plate (250 rpm), bubbled with air, and grown in continuous light (150 μmol/m^2^ s) for 3 days to early stationary phase (~1.2 × 10^6^ cells/mL) before harvesting for DNA isolation. To isolate DNA for methylome analysis, a 2.5 L culture was grown using above conditions to mid exponential phase (5 × 10^5^ cells/mL). On the third day, 300 mL of liquid culture was harvested for the *T* = 0, silicon replete time point for genomic DNA isolation. 1500 mL of cells was harvested by centrifugation and placed into ASW deprived of silica (Si-ASW) as described in [42]. Cells were monitored for 48 h in Si-ASW before harvesting 300 mL DNA for Si-*T* = 48 methylome. Cells were stained for 4,4-difluoro-4-bora-3a,4a-diaza-s-indacene (BODIPY 493/503, Molecular Probes) as described in [42] and imaged using a Zeiss Axio Observer Z1 Inverted Microscope at a 63× objective. Cell growth was monitored using a Neubauer hemocytometer with counts done in duplicate or quadruplicate.

### Functional annotation

Functional annotation of genes from *C. cryptica* and the five other reference diatom genomes was performed using PhyloDB, a comprehensive database of proteins at JCVI. KEGG, KO, KO Pathway, EC annotations were assigned using TimeLogic^®^ Tera-BLAST™ algorithm (Active Motif Inc., Carlsbad, CA, USA), *e* value threshold 1e−5; Pfam/TIGRfam using Hmmer v 3.1b2 (http://hmmer.org), and transmembrane domains with TMHMM 2.0c [87]. Genes from all six genomes were clustered into orthologous groups or larger gene families using MCL [88] with inflation parameter = 2.0. Perl scripts were developed to aggregate cluster annotations based on shared functional content of genes. Methods for prediction of lateral gene transfer, RBH pairs, phylogenetic analysis, and subcellular localization prediction are described in Additional file 1.

## Additional files

**Additional file 1.** Additional methods and figures. **Figure S1.** (a) *Cyclotella cryptica* nuclear assembly overview broken down by minimum contig size (Kbp) versus total Mbp of all contigs (left) and minimum contig size versus the total number of contigs (right) and (b) genomic sequencing data. **Table S1.** Statistics from different gene model prediction pipelines. The pipelines are: (1) FGENESH gene predictions, Diatom training set, (2) Augustus gene predictions V1, *Chlamydomonas* training set, no RNAseq data, (3) Augustus gene predictions V2, FGENESH100 training set, RNAseq data (4) Augustus gene predictions V3, ‘self’ trained, RNAseq data (5) MAKER gene predictions, (Augustus self trained + FGENESH + GeneMarkES). Details are presented in Additional methods. Data presented includes all predicted models regardless of read counts from RNAseq data and prior to removing apparent duplicate contigs (Additional methods, Genome Library Construction and Sequencing). **Table S2.** Repeat sequences in *C. cryptica* identified using (a) RepBase data and (b) RepeatModeler data. **Figure S2.** Phylogenetic comparison of diatom species with sequenced genomes. (a) 18S sequence comparison from [91], accession numbers are listed in Additional file 4. (b) Reciprocal best blast hits comparison. *C. cryptica* and *T. pseudonana* are the most similarly related centric diatoms with available genomic data. **Figure S3.** Per-cytosine fraction methylation. (a) 0 h, silicon replete sample (b) 48 h, silicon-deplete sample. All sites shown have a read coverage greater than or equal to 4. **Figure S4.** Per-site methylation differences between silicon replete and deplete conditions. (a) Histogram showing absolute value of the difference between 48 and 0 h. (b) Scatter plot showing the slope between fraction methylation of genes when comparing the two conditions is linear, outliers are apparent. **Figure S5.** Fraction methylation across the eight largest contigs in *C. cryptica*. Blue is 0 h silicon replete sample, red is 48 h silicon-deplete sample. AUGUSTUS V3 models (labeled “Gene”) are depicted in black while repeat modeler data are depicted in gray. X-axis is number of kilobase pairs in the contig. **Figure S6.** Binned repeats (a) and fraction methylation across gene body (b) in *C. cryptica.* **Table S3.** Summary of gene methylation (AUGUSTUS V3 models) in both experimental conditions. **Figure S7.** Gene methylation relative to gene expression. (a) Three populations emerged (red boxes i–iii) when comparing average fraction methylation to Log2 FPKM. (b) Gene count binned according to FPKM and shaded according to methylation status. Line depicts the proportion of methylated genes per bin. Data shown is for silicon replete, 0 h condition. **Figure S8.** Vector map for ribosomal protein L41 construct for *C. cryptica.*

**Additional file 2.** OrthoMCL and functional annotation, methylome, DarkHorse, subcellular targeting. ND (no data) indicates data not sufficient enough to determine a value.

**Additional file 3.** Periplastid predicted genes.

**Additional file 4.** Triose phosphate transporters, accession numbers for sequences used in Fig. 9 and Additional file 1: Figure S2.

## Abbreviations

AAK14816-like: putative glycerol-3-phosphate acyltransferase
ACC: acetyl-CoA carboxylase
AGPAT: 1-acyl-glycerol-3-phosphate acyltransferase
ASP: Aquatic Species Program
ASW: artificial Seawater
BGS: 1,3 β-glucan synthase
DGAT: diacylglycerol acyltransferase
DGTT: diacylglycerol acyltransferase type 2
ED: Entner Doudoroff
ENO: enolase
ENR: enoyl-ACP reductase
FBA: fructose bisphosphate aldolase
FBP: fructose 1,6 bisphosphatase
Fru6P: fructose-6-phosphate
GAPDH: glyceraldehyde 3-phosphate dehydrogenase
3PG: glycerate 3-phosphate
GLK: glucokinase
GPI: glucose-6-phosphate isomerase
GPAT: glycerol-3-phosphate acyltransferase
HD: 3-hydroxyacyl-ACP dehydratase
KAR: 3-ketoacyl-ACP reductase
KAS: 3-ketoacyl-ACP synthase
LCLAT1: lysocardiolipin acyltransferase 1
LPLAT: lysophospholipid acyltransferase
MAT: malonyl-CoA-ACP transacylase
MDH: malate dehydrogenase
ME: malic enzyme
MGAT: monoacylglycerol acyltransferase
NR: nitrate reductase
OAA: oxaloacetate
PAP: phosphatidic acid phosphatase
PDRP: pyruvate phosphate dikinase regulatory protein
PEP: phosphoenolpyruvate
PEPC: phosphoenolpyruvate carboxylase
PEPCK: phosphoenolpyruvate carboxykinase
PEPS: phosphoenolpyruvate synthase
PFK: phosphofructokinase
PGAM: phosphoglycerate mutase
PGK: phosphoglycerate kinase
PGM: phosphoglucomutase
PK: pyruvate kinase
PPC: periplastid compartment
PPDK: pyruvate phosphate dikinase
PPP: pentose phosphate pathway
PYC: pyruvate carboxylase
RBH: reciprocal best BLAST hit
SAR: Stramenopile, Alveolata, Rhizaria supergroup
TAG: triacylglycerol
TPI: triose phosphate isomerase
UAP: UDP-*N*-acetylglucosamine pyrophosphorylase
UGP: UTP-glucose-1-phosphate uridylyltransferase
