# Supplementary material for: Genome and methylome of the oleaginous diatom Cyclotella cryptica reveal genetic flexibility toward a high lipid phenotype
Source: Biotechnol Biofuels. 2016 Nov 25;9:258. doi: 10.1186/s13068-016-0670-3 (PMC5124317; doi:10.1186/s13068-016-0670-3)

Supplemental Methods for Traller et al "Genome and methylome of the oleaginous diatom *Cyclotella cryptica* reveal genetic flexibility toward a high lipid phenotype"

DNA extraction and purification

Liquid cell cultures concentrated either by filtration using a 3.0μm polycarbonate filter (DNA for genome sequencing) or by centrifugation in 50mL conical tubes at 4000 xg for 8 minutes (methylome DNA) using in an Eppendorf 5810R centrifuge. DNA for genome and bisulfite sequencing was purified using CsCl as described in [1]. To remove RNA contamination, DNA for bisulfite sequencing was treated with 10mg/mL stock of RNase A for 15 minutes at 37ºC.

Genome Library Construction and Sequencing

Three libraries were prepared following Illumina's standard genomic DNA paired end construction: "PE-short" (post-assembly empirical insert lengths ~90-235 nucleotides (nt) exclusive of adapters [preparation target 180-230 nt], with mode 123 nt), "PE-medium" (~155-330 nt [preparation target 230- 330 nt], mode 221 nt), and "PE-long" (~225-460 nt [preparation target 330-430 nt] with mode 305 nt) for ~58% of inserts, with the balance in a second mode ~60-225 nt peaking at ~105 nt). These were sequenced as paired end 76-mer + 76-mer reads on an Illumina GA-IIx 120-tiles/lane run ("TP003") at the UCLA BSCRC Core Sequencing facility, using two dedicated lanes for each of PE-short and PE-medium, three dedicated lanes for PE-long, a dedicated PhiX control lane for RTA image analysis autocalibration, and spiking in ≈1% Illumina PhiX into each non-control lane. Two genomic DNA mate pair libraries (aiming for 10K nt effective inserts) were prepared and run by Illumina service on a 48-tile/lane v3 HiSeq flow cell, each library on

a single dedicated lane: "MP-short" (effective ~2,100-3,320 nt with mode ~2,625 nt for ~58% of inserts, with the balance in a PE-orientation [rather than MP-orientation] second mode ~170-480 nt peaking at ~205 nt), and "MP-long" (~1,740-2,730 nt with mode ~2,260 nt for ~63% of inserts, with the balance in a PE-oriented second mode ~176-535 nt peaking at ~223 nt). These were physically sequenced as paired end 101-mer + 10-mer index + 101-mer, with the index reads and the last base of each main end discarded (in the usual way so that the last retained base has bidirectional RTA phasing corrections). The number of raw read pairs for PE-short/medium/long/control is found in Additional File 1, Figure S1b. Only read pairs with no 'N' basecalls were retained; due to the pattern of 'N' basecalls in PE-long, the first four bases of each end of its lanes were discarded before this filter (and subsequent uses of this library).

The number of raw read pairs for MP-short and long libraries was 142,455,072 and 154,107,079, respectively, and only RTA PF=1-passing pairs were retained (Additional File 1, Figure S1b). Although not used as a filter, relative to PF=1 read pairs, the 'N'-free read pairs for MP-short/long were ~98.9% / 98.6%.

The PE and MP libraries contributed ~23.4G nt and ~57.4G nt, respectively, for a total of ~80.8G nt (≈461x coverage of a 175Mbp genome; for 65-mers: ~3.4G and ~20.7G, total ~24.0G and ≈137x).

Using many iterations of a variety of standard and *ad hoc* assemblers and alignment tools, with extensive inspection of intermediate stages and judgment calls made by hand, read pairs from PE-short/medium/long and MP-short/long were used to construct high-quality best assemblies from the available data for the chloroplast ("chrC") and mitochondrial ("chrM") genomes in *C. cryptica*. In each case, a single complete circular

sequence of pure A/C/G/T's without gaps was formed (chrC 129,320 bp, chrM 58,021 bp). This was greatly assisted by the presence in NCBI of related genomes: KJ958480.1 for *Cyclotella* strain L04_2 chrC (~96% identity; also useful: KJ958481.1 for *Cyclotella* strain WC03_2 chrC), and NC_007405.1 for *Thalassiosira pseudonana* chrM (more distant; even on homologous stretches, overall percent identity ≈80%). The *C. cryptica* chrM is estimated with 17,880 bp (~31%) being 120 exact copies of the 149 bp sequence
TTATCGGCCTCAAATCAAGCAGTGTTTAAGCTGGAAT
CTATCGGCCTCAAATCGAAACAGTGTTTTAGCCTGAAT
TTATCGGCCTCAAATCAAGCAGTGTTTAAGCTGGAAT
CTATCGGCCTCAAATCGAAACAGTGTTTTGCCTGAAT (which is itself four approximate tandem copies of a smaller unit). Given current data, this region cannot be completely resolved, and there is likely additional variation here, and the included number of copies is an estimate informed in part by depth of coverage relative to other chrM sequence.

The main genome assembly was performed with an ABySS 1.3.1 SE+PE+MP pipeline using k=65-mers, t=65, q=3, e=3, E=0, c=3, m=30, p=0.9, no scaffolding at PopBubbles, s=200, n=10, overlap min=5 with scaffolding and join masking at simple repeats, SimpleGraph d=6 and scaffolding, greedy MergePaths, a=4, and abyss-scaffold min-gap=100. The SE stage used PE-short+medium+long, MP-short+long, the PE stage was applied to PE-short+medium+long, and the MP stage was applied to MP-short+long. The assembly was taken as the final scaffolds of nt length $\geq 130 = 2k$. Based on alignments, scaffolds apparently consisting of PhiX, chrC, or chrM were removed.

Genome annotation revealed several nearly identical genomic contigs with nearly identical fold coverage, which appear to be due to a failure of the assembler to collapse identical contigs into one contig. In an effort to resolve this artifact from the assembler, we performed all versus all BLASTn for each genomic contig against the whole genome and removed contigs which contained a >95% threshold for query coverage and nucleotide identity. This reduced the total number of contigs from 199,501 to 116,817 (41.3% reduction) and total genome size from 182,854,974bp to 161,759,242bp (excluding the mitochondrial and chloroplast genomes).

Notation for contigs is in the form of gXXXXXX_YYYYY, where XXXXXX gives contig length and YYYYY gives the approximate average coverage for that contig. Contigs beginning with a 'g' are genomic sequences and contigs beginning with an 'r' are mRNA sequences.

DNA Bisulfite sequencing and analysis

1 μg of *C. cryptica* nuclear DNA for bisulfite treatment was resuspended in 50 μl of EB buffer (QIAGEN) and sonicated in AFA-fiber microTubes using a Covaris S2 machine (Duty Cycle = 10%; Intensity = 5; Cycles/Burst = 200; for 6 minutes) to obtain 100-300 bp fragments. The DNA was then subjected to End-Repair, A-tailing and Adapter Ligation using Illumina TruSeq DNA Sample Prep kit v2 according to manufacturer's instructions. The Adapter-ligated DNA was purified and size-selected using AMPure XP beads. DNA was then bisulfite treated using EpiTect kit (QIAGEN) using the following conversion protocol: 95°C 5min, 60°C 25min, 95°C 5min, 60°C 85min, 95°C 5min, 60°C 175min, 95°C 5min, 60°C 25min, 95°C 5min, 60°C 85min,

95°C 5min, 60°C 175min. Bisulfite-treated DNA was then desulphonated according to manufacturer's protocol ("Small Amount of Fragmented DNA" variant) and DNA eluted twice with EB. Converted DNA was amplified with MyTaq 2x mix (Bioline): 98°C 2 min; 12 cycles of 98°C 15 sec, 60°C 30 sec, 72°C 30 sec; 72°C 5 min. Amplified DNA was diluted to 10 nM and sequenced using Illumina HiSeq2000 (100 single end reads).

Bisulfite converted reads were inspected for sequencing quality using FastQC 0.10.1. Reads passing the Illumina quality filter ('PF' value equal to 1) were retained and aligned to the genome assembly using BS-Seeker2 in local alignment mode with Bowtie2 as the aligner [2]. For the purpose of this study, a 'methylated' base pair is defined as a cytosine which has >= 4 methylated reads, similar to as described in [3]. Therefore an 'unmethylated' cytosine is that which has >= 4 unmethylated reads. Those cytosines which do not have at least 4 aligned reads from the bisulfite sequencing are considered to not have enough data sufficiently conclude whether that site is methylated or not. Genes that are 'methylated' are those which are defined as having >=50% of callable CG sites (the most common motif for methylation) containing a 'methylated' cytosine. Unmethylated genes are <50%.

RNA sequencing

Total RNA was purified from cultures of *C. cryptica* in log-phase growth under conditions of either silicon starvation or nitrogen starvation. For RNA isolation, 750mL of liquid cell culture for each time point was harvested and treated with 20mg/mL cycloheximide and concentrated by filtration. Cells were stored in -80ºC prior to extraction. Total RNA was extracted according to [4,5].

Five µg of total RNA per sample was treated with Turbo DNase (Ambion) for 30 min at 37°C according to the manufacturer's instructions in order to remove any contaminating DNA. The resulting RNA was purified by ethanol precipitation, and RNA quality was evaluated on a BioAnalyzer RNA Nano kit (Agilent). RNAseq libraries were prepared using the Illumina TruSeq mRNA Sample Prep kit (Illumina) according to manufacturer's protocols (Rev. A). Sequencing was performed at the UCLA Broad Stem Cell Research Center sequencing core on a HiSeq 2000 sequencer (Illumina) using a mixture of 50+50 nt paired end reads and 100 nt single end reads. 11 libraries were sequenced on a single lane each, 2 libraries were multiplexed onto a 1 lane. Pooled raw reads were demultiplexed and all reads mapped to transcriptome data, with remaining unmapped reads mapped to reference *Cyclotella* genome version cycCry0dot2 using TopHat 2.0.4 allowing for two mismatches, reporting only unique mappings [6]. Bam files were processed through HTSeq 0.5.3 using the "intersection-nonempty" method which assigns a read to a gene only if the read overlaps with only one gene in its entirety. Single-end and paired-end data were combined and all data was run through DESeq 1.8.3 to obtain FPKM counts [7]. A combination of 50+50 nt paired end reads and 100 nt single end reads were pooled to facilitate accurate mapping of the genes.

Genome Annotation

Gene model predictions were generated from several pipelines as follows: (1) FGENESH using the built-in diatom training set; (2) standalone AUGUSTUS using the built-in '*Chlamydomonas reinhardtii'* training parameters; (3) standalone AUGUSTUS using the 100 longest FGENESH predictions as a training set; (4) web-based

AUGUSTUS trained on the de-novo RNA assembly; and (5) MAKER with FGENESH, AUGUSTUS, GeneMarkES analyses enabled [8-11]. All prediction software was run using default settings except where noted. Several genes were selected where intron/exon boundaries were well characterized in *T. pseudonana* and used to test the accuracy of the gene model predictions. AUGUSTUS predictions from set (4) that overlap MAKER predictions from set (5) constitute the 'high-confidence' collection of gene predictions. For gene structure, it was determined that (4) more accurately predicted start/stop sites and intron/exon boundaries. Gene set (4 and 5) were functionally annotated [12].

Repetitive elements were identified using RepeatMasker 4.0.1 with RMBLASTN 2.2.27+, using the Bacillariophyta repeat library from RepBase and default settings. Additional repeat masking by RepeatMasker was performed using a custom de-novo library generated using RepeatModeler 1.0.7 with the NCBI BLAST engine.

The diatom genomes used in Figure 9 and S2, OrthoMCL, and RBH analyses are *T. pseudonana* v3.0 [13], *P. tricornutum* v2.0 [14], *F. cylindrus* v1.0 [15], *P. multiseries* v1.0 [16], and *T. oceanica* v.3.0 (NCBI accession numbers JP288099-JP2977110, http://www.ncbi.nlm.nih.gov)

For reciprocal best BLAST hit (RBH) analysis, gene models from diatom genomes (listed above) were aligned using BLASTp 2.2.28+ [17], with an e-value cutoff score of 1e-5 and a query coverage of at least 70%. RBH pairs were detected using python script [18].

Predicted proteins from *T. pseudonana* and *C. cryptica* genomes were evaluated for phylogenetic relatedness to sequences in NCBI GenBank nr (accessed October 16, 2015)

using the DarkHorse program version 1.5 with a threshold filter setting of 0.1 [24]. BLASTP alignments to GenBank nr sequences were required to cover at least 70% of total query length and have e-value scores of $1e^{-5}$ or better for inclusion in this analysis.

For phylogenetic analysis in Figure 9 and S2, and to further investigate proteins of interest, amino acid sequences were aligned using MUSCLE with 10 maximum number of iterations and default parameters [19]. Trees were generated using default parameters in RAxML_GUI v1.3 for 100 iterations and visualized using FigTree v1.4.0 [20]. For each tree shown, bootstrap values are listed and have been midpoint rooted.

Subcellular Localization Prediction

All open reading frames were analyzed using several computational tools for predicting the likely location of the protein product within the cell. Nucpred [21] and NetNES [22] provided estimations of nuclear localization for each putative protein. PREDOTAR [23], PSORT [24] , HECTAR [25], CELLO [26] , PredAlgo [27], SignalP 3.0 [28] TargetP, ChloroP [29] and ASAFind [30] provided possible localizations to mitochondria, chloroplast, endoplasmic reticulum, or plastid. The presence of N-terminal signal peptides and transmembrane regions were assessed using PHOBIUS [31,32], the SignalP 3.0 portion of TargetP and PredAlgo. Specific sequences and predicted cleavage sites for signal peptides were taken from SignalP 3.0. All open reading frames from *C. cryptica* were submitted to web-servers in an automated fashion using scripts for html or webmail submission.

Methods for targeting predictions shown in Figure 4 are as follows: for plastid targeting, all proteins with predicted positive ASAfind plastid targeting were surveyed for

proper cleavage site using SignalP 3.0 [28, 30]. Predicted SignalP 3.0 cleavage sites were then defined by the guidelines addressed in Figure 5 of [33] and split into canonical plastid (AF, GF, and SF cleavage sites), noncanonical plastid (AW, AY, AL, GW, GY, GL, SW, SY, SL), periplastid and other (AH, AI, AM, AR, AE, AG, SH, SI, SM, SR, SE, SG, GH, GI, GM, GR, GE, GG). Periplastid predicted cleavage sites which did not contain a positive ChloroP prediction were then categorized as ER/secreted. Any negative plastid ASAfind result but SignalP 3.0 positive with a predicted periplastid cleavage site, and positive ChloroP were also classified as periplastid. ER/secreted proteins were then defined as proteins with negative plastid ASAfind prediction, positive SignalP 3.0, with a cleavage site not one that is specified in [33].

Mitochondrially-targeted proteins were classified according to any of the following prediction combinations for HECTAR, Predotar, and TargetP (listed in respective order): Type II, Mito, Mito; SigP, Mito, Mito; Mito, Plastid, Mito; Mito, Mito, SigP; Mito, Mito, Mito; Mito, Mito, Plastid; Mito, Mito, no prediction; Mito, no prediction, Mito; no prediction, Mito, Mito. MitoProt was also used for all predicted proteins shown in Figure 5-8 [34]. All other proteins that did not fall into the above criterion were classified as 'cytosol and other.'

Vector Construction and Diatom Transformation

The vector utilizing the *C. cryptica* rpL41 promoter and terminator sequences was assembled using the GeneArt® Seamless Cloning and Assembly Kit (Invitrogen), with a Gateway™ vector (Invitrogen) as the backbone. The construction of the *T. pseudonana fcp* [35] and nitrate reductase (NR) [36,37] vectors is described elsewhere.

Each vector was co-transformed with another vector expressing the *nat1* gene (received from N. Kroger, Germany), which confers resistance to the antibiotic nourseothricin, under control of the acetyl coenzyme A carboxylase promoter. *C. cryptica* was transformed using tungsten microparticle bombardment as described elsewhere (Shrestha and Hildebrand, 2014), with the following changes: $1x10^7$ cells in exponential phase were spread onto artificial sea water (ASW) containing 1.5% Bacto Agar (Becton Dickinson, USA) and no antibiotics [38]. Immediately after bombardment, 10ml ASW was added to the plates, which were incubated in low light for 18 hours. Following the incubation, cells were washed off the plates using the ASW they were incubated with, and the entire volume was transferred to 125ml Erlenmyer flasks with 50ml ASW with 100μg/ml nourseothricin (clonNAT; Werner BioAgents, Germany). After 7 days of growth with shaking, 10ml of the cultures were transferred to 125ml Erlenmeyer flasks with fresh 50ml ASW plus 100μg/ml nourseothricin and grown to exponential phase. The highest GFP expressors were selected using a sorting flow cytometer (Influx, Becton Dickinson, USA), recovered in liquid ASW for 2 days, then plated on ASW agar plates with 100μg/ml nourseothricin. Colonies were picked and transferred to 24 well plates, each well containing 2 ml ASW with 100μg/ml nourseothricin.

Assessment of Conditional Expression Using the Nitrate Reductase Promoter

Three independent clones were grown to exponential phase in 24 well plates, as described above. 2μl of culture was transferred to two new wells per clone. One of the wells contained 2ml ASW, which would allow for expression of GFP under the NR promoter. The other well contained 2ml modified ASW, in which ammonia replaced

nitrate as the nitrogen source, which would repress the expression of GFP under the NR promoter [36]. The cultures were allowed to reach exponential phase and imaged using Zeiss Axio Observer Z1 Inverted Microscope (Zeiss Axioscope, Carl Zeiss Microimaging Inc., USA). The filter set for fluorescent imaging was Emission LP 515 nm for chlorophyll autofluorescence, and Zeiss #38HE Excitation BP 470/40 nm, Dichromatic mirror FT 495 nm, Emission BP 525/50 nm for GFP.

1. Jacobs JD, Ludwig JR, Hildebrand M, Kukel A, Feng T-Y, Ord RW, et al. Characterization of two circular plasmids from the marine diatom Cylindrotheca fusiformis: plasmids hybridize to chloroplast and nuclear DNA. Molec. Gen. Genet. 1992;233:302–10.

2. Guo W, Fiziev P, Yan W, Cokus S, Sun X, Zhang MQ, et al. BS-Seeker2: a versatile aligning pipeline for bisulfite sequencing data. BMC Genomics. 2013;14:774.

3. Lopez DA, Hamaji T, Kropat J, De Hoff P, Morselli M, Rubbi L, et al. Dynamic changes in the transcriptome and methylome of Chlamydomonas reinhardtii throughout its life cycle. plantphysiol.org. American Society of Plant Biologists; 2015;169:00861.2015–743.

4. Smith SR, Glé C, Abbriano RM, Traller JC, Davis A, Trentacoste E, et al. Transcript level coordination of carbon pathways during silicon starvation‐induced lipid accumulation in the diatom Thalassiosira pseudonana. New Phytol. 2016;210:810-904.

5. Hildebrand M, Dahlin K. Nitrate transporter genes from the diatom Cylindrotheca fusiformis (Bacillariophyceae): mRNA levels controlled by nitrogen source and by the cell cycle. J. Phycol. 2000;36:702-13.

6. Kim D, Pertea G, Trapnell C, Pimentel H, Kelley R. TopHat2: accurate alignment of transcriptomes in the presence of insertions, deletions and gene fusions. Genome Biol. 2013;14:R36.

7. Love MI, Huber W, Anders S. Moderated estimation of fold change and dispersion for RNA-seq data with DESeq2. Genome Biol. 2014;15:550.

8. Stanke M, Morgenstern B. AUGUSTUS: a web server for gene prediction in eukaryotes that allows user-defined constraints. Nucleic Acids Res. 2005;33:W465–7.

9. Cantarel BL, Korf I, Robb SMC, Parra G, Ross E, Moore B, et al. MAKER: An easy-to-use annotation pipeline designed for emerging model organism genomes. Genome Res. 2007;18:188–96.

10. Salamov AA. Ab initio Gene Finding in Drosophila Genomic DNA. Genome Res. 2000;10:516–22.

11. Borodovsky M, Lomsadze A. Eukaryotic gene prediction using GeneMark.hmm-E and GeneMark-ES. Curr Protoc Bioinformatics. 2011;Chapter 4:Unit4.6.1–10.

12. Lopez D, Casero D, Cokus SJ, Merchant SS, Pellegrini M. Algal Functional Annotation Tool: a web-based analysis suite to functionally interpret large gene lists using integrated annotation and expression data. BMC Bioinformatics. 2011;12:282.

13. Joint Genome Institute. http://genome.jgi.doe.gov/Thaps3/Thaps3.home.html

14. Joint Genome Institute. http://genome.jgi.doe.gov/Phatr2/Phatr2.home.html

15. Joint Genome Institute. http://genome.jgi.doe.gov/Fracy1/Fracy1.home.html

16. Joint Genome Institute. http://genome.jgi.doe.gov/Psemu1/Psemu1.home.html

17. Altschul SF, Gish W, Miller W, Myers EW. Basic local alignment search tool. J. Mol. Biol. 1990;215:403-10.

18. Qin H. hongqin/Simple-reciprocal-best-blast-hit-pairs.

19. Edgar RC. MUSCLE: multiple sequence alignment with high accuracy and high throughput. Nucleic Acids Research. 2004;32:1792–7.

20. Stamatakis A. RAxML-VI-HPC: maximum likelihood-based phylogenetic analyses with thousands of taxa and mixed models. Bioinformatics. 2006;22:2688–90.

21. Brameier M, Krings A, MacCallum RM. NucPred Predicting nuclear localization of proteins. Bioinformatics. 2007;23:1159–60.

22. La Cour T, Kiemer L, Mølgaard A, Gupta R, Skriver K, Brunak S. Analysis and prediction of leucine-rich nuclear export signals. Protein Engineering Design and Selection. 2004;17:527–36.

23. Small I, Peeters N, Legeai F, Lurin C. Predotar: A tool for rapidly screening proteomes forN-terminal targeting sequences. Proteomics. 2004;4:1581–90.

24. Horton P, Park KJ, Obayashi T, Fujita N, Harada H, Adams-Collier CJ, et al. WoLF PSORT: protein localization predictor. Nucleic Acids Res. 2007;35:W585–7.

25. Gschloessl B, Guermeur Y, Cock JM. HECTAR: A method to predict subcellular targeting in heterokonts. BMC Bioinformatics. 2008;9:393.

26. Yu C-S, Lin CJ, Hwang J-K. Predicting subcellular localization of proteins for Gram-negative bacteria by support vector machines based on n-peptide compositions. Protein Science. Cold Spring Harbor Laboratory Press; 2004;13:1402–6.

27. Tardif M, Atteia A, Specht M, Cogne G, Rolland N, Brugiere S, et al. PredAlgo: A New Subcellular Localization Prediction Tool Dedicated to Green Algae. Mol Biol Evol. 2012;29:3625–39.

28. Dyrløv Bendtsen J, Nielsen H, Heijne von G, Brunak S. Improved Prediction of Signal Peptides: SignalP 3.0. J Mol Biol. 2004;340:783–95.

29. Emanuelsson O, Brunak S, Heijne von G, Nielsen H. Locating proteins in the cell using TargetP, SignalP and related tools. Nat Protoc. 2007;2:953–71.

30. Gruber A, Rocap G, Kroth PG, Armbrust EV, Mock T. Plastid proteome prediction for diatoms and other algae with secondary plastids of the red lineage. Plant J. 2015;81:519–28.

31. Laws EA, Taguchi S, Hirata J, Pang L. Optimization of microalgal production in a shallow outdoor flume. Biotechnol. Bioeng. 1988;32:140–7.

32. Käll L, Krogh A, Sonnhammer E. A Combined Transmembrane Topology and Signal Peptide Prediction Method. J Mol Biol. 2004;338:1027–36.

33. Gruber A, Vugrinec S, Hempel F, Gould SB, Maier U-G, Kroth PG. Protein targeting into complex diatom plastids: functional characterisation of a specific targeting motif. Plant Mol Biol. 2007;64:519–30.

34. Claros MG, Vincens P. Computational method to predict mitochondrially imported proteins and their targeting sequences. Eur J Biochem. 1996.

35. Shrestha RP, Hildebrand M. Evidence for a Regulatory Role of Diatom Silicon Transporters in Cellular Silicon Responses. Eukaryot Cell. 2014;14:29–40.

36. Poulsen N, Chesley PM, Kröger N. Molecular genetic manipulation of the diatom Thalassiosira pseudonana (Bacillariophyceae). J. Phycol. 2006;42:1059–65.

37. Trentacoste EM, Shrestha RP, Smith SR, Glé C, Hartmann AC, Hildebrand M, et al. Metabolic engineering of lipid catabolism increases microalgal lipid accumulation without compromising growth. Proc Nat Acad Sci. 2013;110:19748–53.

38. Darley WM, Volcani BE. Role of silicon in diatom metabolism. Exp Cell Res. 1969;58:334–42.

Figure S1: (a) *Cyclotella cryptica* nuclear assembly overview broken down by minimum contig size (Kbp) versus total Mbp of all contigs (left) and minimum contig size versus the total number of contigs (right) and (b) genomic sequencing data.

a.

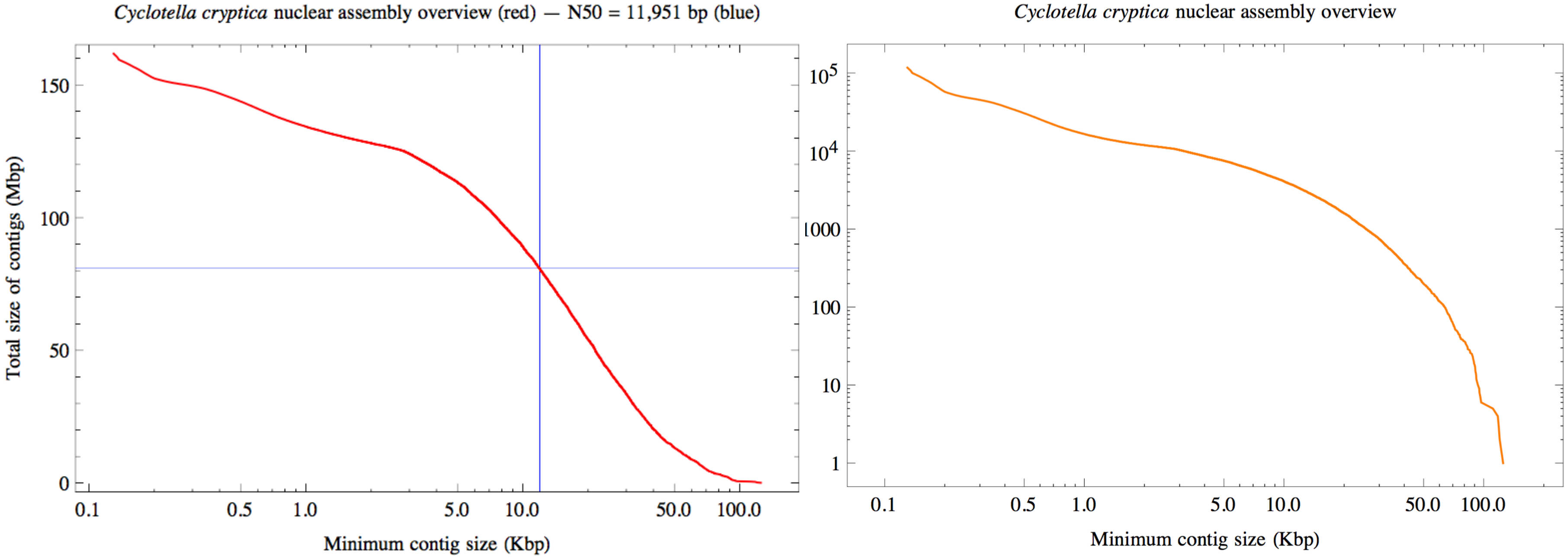

b.

Genomic Libraries	Number of Raw Reads for Paired End Libraries				Percentage passing pairs				Percentage passing pairs after removal of pairs with 'N'
	Lane 1	Lane 2	Lane 3	Total Reads	Lane 1	Lane 2	Lane 3	Average	Average
Paired End Short	65270867	17592293		82863160	4.8	57		71.1	73.0
Paired End Medium	65333130	66235838		131568968	68.5	69.5		60.0	79.6
Paired End Long	69147985	69578501	65179466	203905952	27.8	34	65.6	42.5	57.9
Paired End Control	30301553				92.8				99.5
Mate Pair Short	142455072				96.9				98.9
Mate Pair Long	154107079				96.7				98.6

Supplementary table S1. Statistics from different gene model prediction pipelines. The pipelines are: (1) FGENESH Gene predictions, Diatom training set, (2) Augustus Gene predictions V1, *Chlamydomonas* training set, no RNAseq data, (3) Augustus Gene predictions V2, FGENESH100 training set, RNAseq data (4) Augustus Gene predictions V3, 'self' trained, RNAseq data (5) MAKER Gene predictions, (Augustus self trained + FGENESH + GeneMarkES). Details are presented in Supplementary Methods. Data presented includes all predicted models regardless of read counts from RNAseq data and prior to removing apparent duplicate contigs (Additional File 1: Supplementary Methods, Genome Library Construction and Sequencing).

	**Gene Model Prediction Pipeline**				
	**CcFgenesh**	**CcAugustusV1**	**CcAugustusV2**	**CcAugustusV3**	**CcMAKERV1**
**Total Models**	50,288	6,295	24,819	**33,682**	**9,049**
**Average Model Length (bp)**	1,561.8	926.1	1,746.2	**1,265**	**1,999.7**
**Average Exons**	3.72	1.22	2.65	**1.95**	**3.69**
**Avg. Exon Length (bp)**	247.4	115.8	561.5	**585.6**	**457.6**
**Avg. # Introns**	2.72	0.22	1.65	**0.95**	**2.69**
**Avg. Intron Length (bp)**	129.8	542	153.9	**128.1**	**128.1**

Table S2: Repeat sequences in *C. cryptica* identified using (a) RepBase data and (b) RepeatModeler Data.

a.

Repeat sequences in *Cyclotella cryptica* using RepBase data
Sequences: 199501
total length: 182854974 bp (174198679 bp excl N/X-runs)
GC level: 43.01%
Bases masked: 7037708 bp (3.85 %)

	Number of elements*	Length occupied (bp)	Percentage of sequence
**Retroelements**	12264	4694900	2.57
**SINEs:**	0	0	0
**Penelope**	13	1650	0
**LINEs:**	58	4854	0
**CRE/SLACS**	0	0	0
**L2/CR1/Rex**	0	0	0
**R1/LOA/Jockey**	0	0	0
**R2/R4/NeSL**	0	0	0
**RTE/Bov-B**	2	100	0
**L1/CIN4**	0	0	0
**LTR elements:**	12206	4690046	2.56
**BEL/Pao**	0	0	0
**Ty1/Copia**	2901	1432661	0.78
**Gypsy/DIRS1**	9305	3257385	1.78
**Retroviral**	0	0	0
**DNA transposons**	346	113716	0.06
**hobo-Activator**	0	0	0
**Tc1-IS630-Pogo**	0	0	0
**En-Spm**	0	0	0
**MuDR-IS905**	0	0	0
**PiggyBac**	2	103	0
**Tourist/Harbinger**	305	105959	0.06
**Other (Mirage, P-element, Transib)**	0	0	0
**Rolling-circles**	0	0	0
**Unclassified:**	28	6197	0
**Total interspersed repeats:**		4814813	2.63
**Small RNA:**	142	17166	0.01
**Satellites:**	0	0	0
**Simple repeats:**	32133	1928189	1.05
**Low complexity:**	3624	294614	0.16

b.

Repeat sequences in *Cyclotella cryptica* using RepeatModeler data
Sequences: 199501
Total length: 182854974 bp (174198679 bp excl N/X-runs)
GC level: 43.01 %
Bases masked: 98288109 bp (53.75 %)

	Number of elements*	Length occupied (bp)	Percentage of sequence
**SINEs:**	0	0	0
**ALUs**	0	0	0
**MIRs**	0	0	0
**LINEs:**	2626	1877047	1.03
**LINE1**	0	0	0
**LINE2**	0	0	0
**L3/CR1**	0	0	0
**LTR elements:**	43176	15802661	8.64
**ERVL**	0	0	0
**ERVL-MaLRs**	0	0	0
**ERV_classI**	0	0	0
**ERV_classII**	0	0	0
**DNA elements:**	15402	5899536	3.23
**hAT-Charlie**	0	0	0
**TcMar-Tigger**	155	44023	0.02
**Unclassified:**	314059	73067346	39.96
**Total interspersed repeats:**		96646590	52.85
**Small RNA:**	0	0	0
**Satellites:**	0	0	0
**Simple repeats:**	23628	1861243	1.02
**Low complexity:**	1672	129011	0.07

Figure S2: Phylogenetic comparison of diatom species with sequenced genomes. (a) 18S sequence comparison from [89], accession numbers are listed in Additional File 4 (b) Reciprocal best blast hits comparison. *C. cryptica* and *T. pseudonana* are the most similarly related centric diatoms with available genomic data.

a.

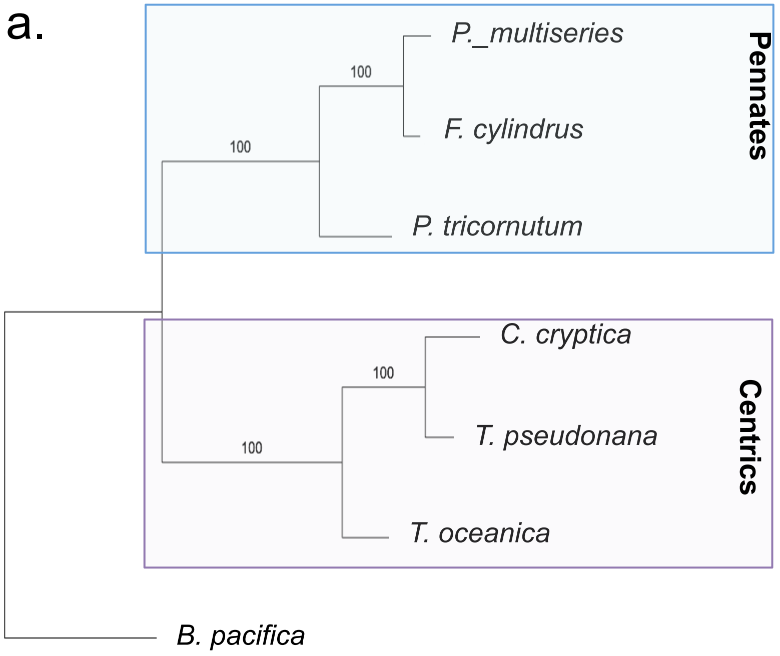

b.

	Total Reciprocal BLAST Hits	Average % Identity
C. cryptica/T. pseudonana	5498	63.6
C. cryptica/T. oceanica	4443	56.4
T. pseudonana/T. oceanica	3592	59.2
P. tricornutum/F. cylindrus	7060	55.5
P. tricornutum/P. multiseries	2878	54.2
F. cylindrus/P. multiseries	7773	66.0

Figure S3: Per-cytosine fraction methylation. (a) 0 hour, silicon-replete sample (b) 48 hour, silicon-deplete sample. All sites shown have a read coverage greater than or equal to 4.

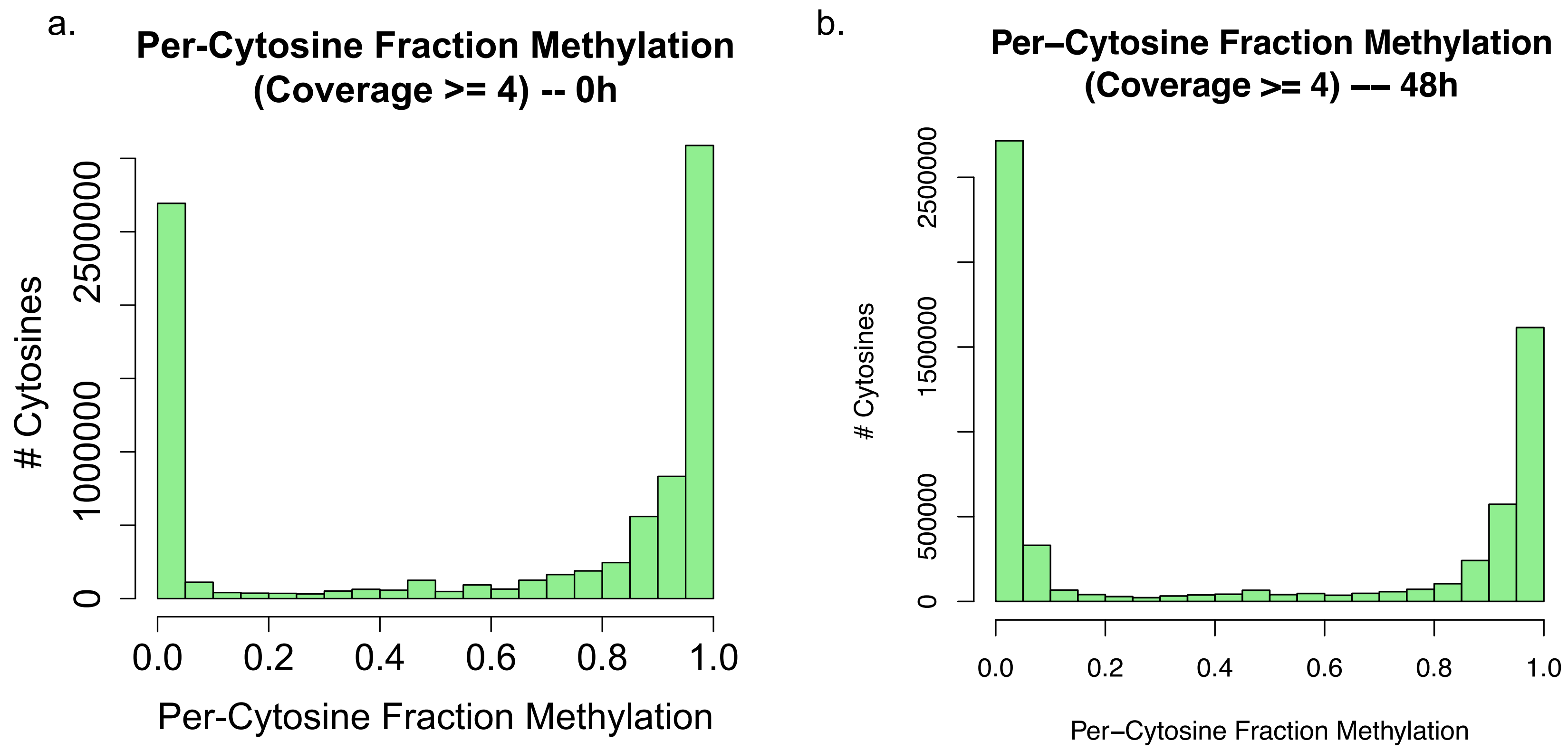

Figure S4: Per-site methylation differences between Silicon replete and deplete conditions. (a) Absolute value of the difference between 48h and 0h. (b) Slope between fraction methylation of genes in when comparing the two conditions is linear, outliers are apparent.

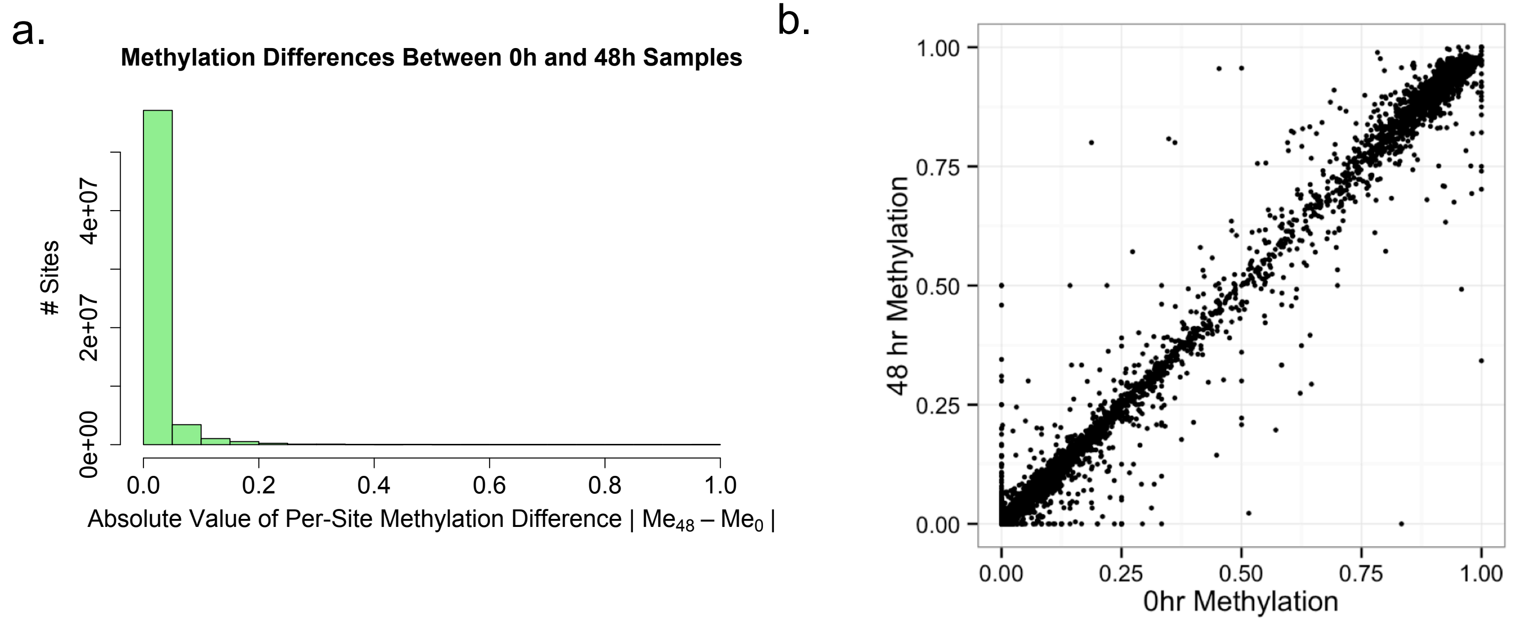

Figure S5: Per-site methylation differences between silicon replete and deplete conditions. (a) Histogram showing absolute value of the difference between 48h and 0h. (b) Scatter plot showing the slope between fraction methylation of genes in when comparing the two conditions is linear, outliers are apparent.

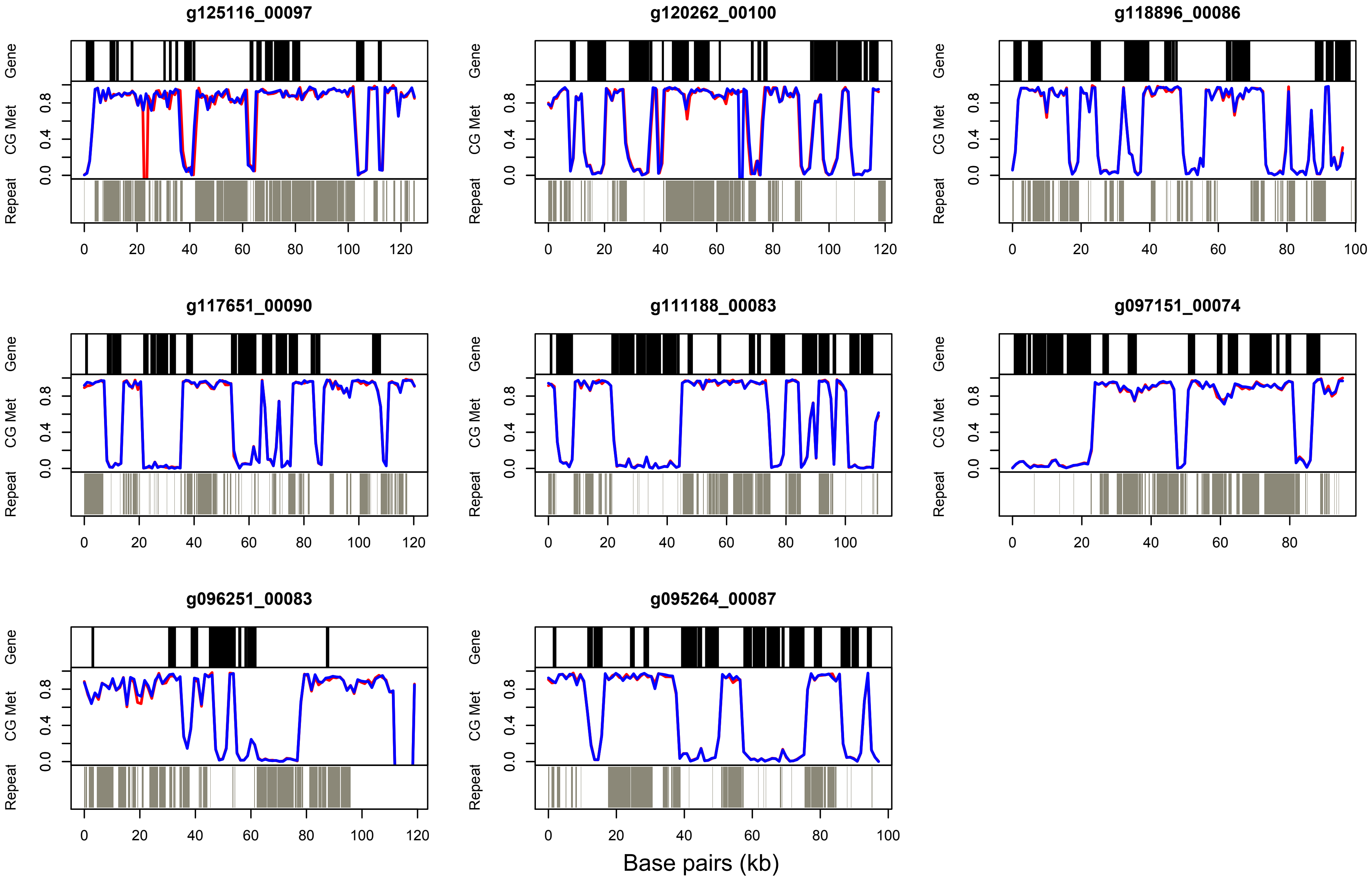

Figure S6: Binned repeats (a) and fraction methylation across gene body (b) in *C. cryptica.*

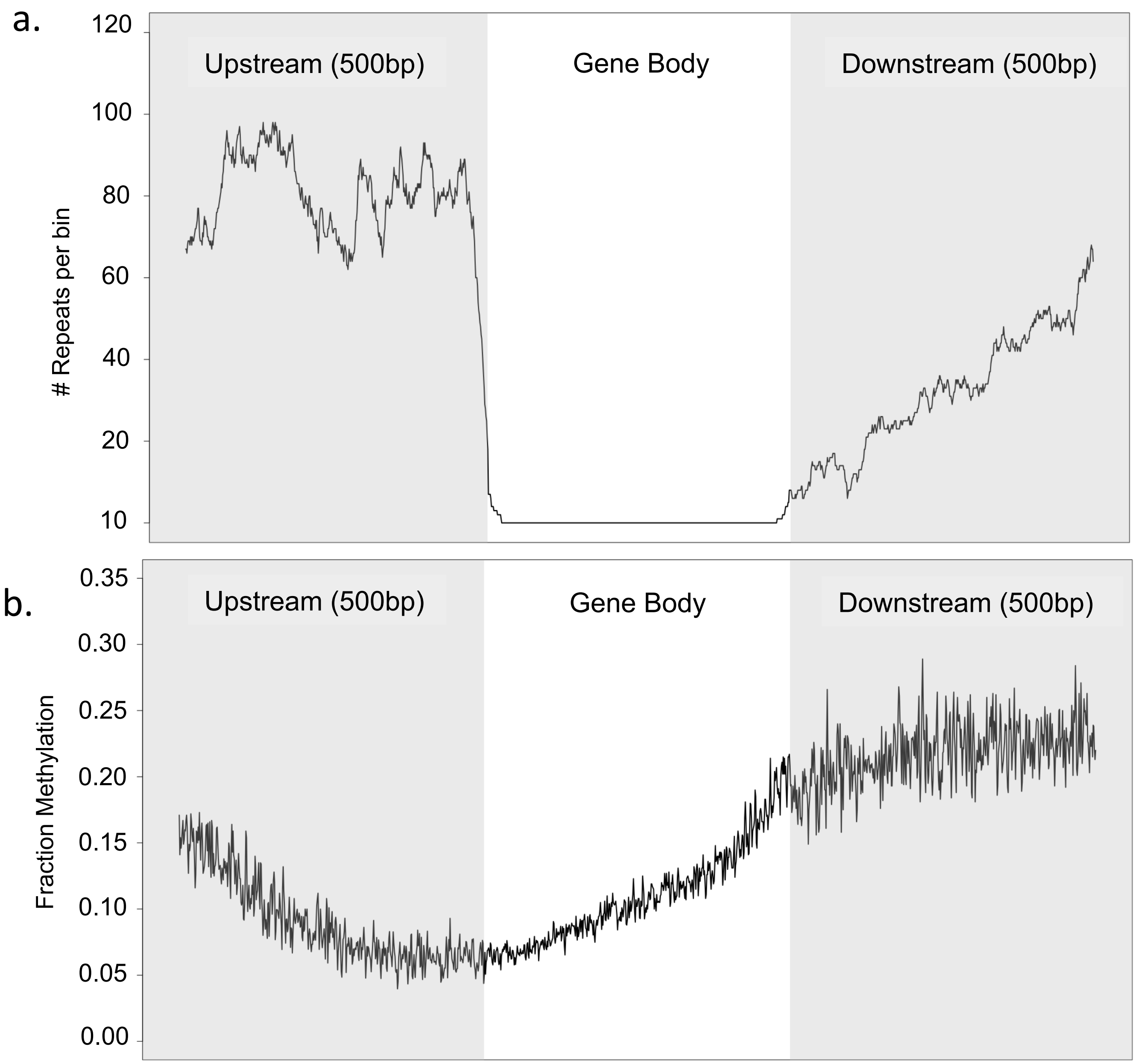

Table S3: Summary of gene methylation (AUGUSTUS V3 models) in both experimental conditions.

Silicon Replete 0hr					
	Count	Percent total of all genes	Percent total Excluding genes with insufficient coverage	Average Fraction Methylation	Average FPKM
Methylated genes	4170	20	22	0.87	536
Unmethylated genes	14866	70	78	0.07	4593
No Methylation Data	2085	10	-	ND	1767
Total genes	21121			0.24	3520
**Silicon Deplete 48hr**					
	Count	Percent total of all genes	Percent total Excluding genes with insufficient coverage	Average Fraction Methylation	Average FPKM
Methylated genes	2627	12	24	0.88	523
Unmethylated genes	8453	40	76	0.07	4589
No Methylation Data	10041	48	-	ND	3641
Total genes	21121			0.26	3408

	Methylated 48hr	Unmethylated 48hr
**Methylated 0hr**	2584	31
**Unmethylated 0hr**	17	8165

Figure S7: Gene methylation relative to gene expression. (a) 3 populations emerged (Red boxes i-iii) when comparing average fraction methylation to Log2 FPKM. (b) Gene count binned according to FPKM and shaded according to methylation status. Line depicts the proportion of methylated genes per bin. Data shown is for silicon replete, 0 hour condition.

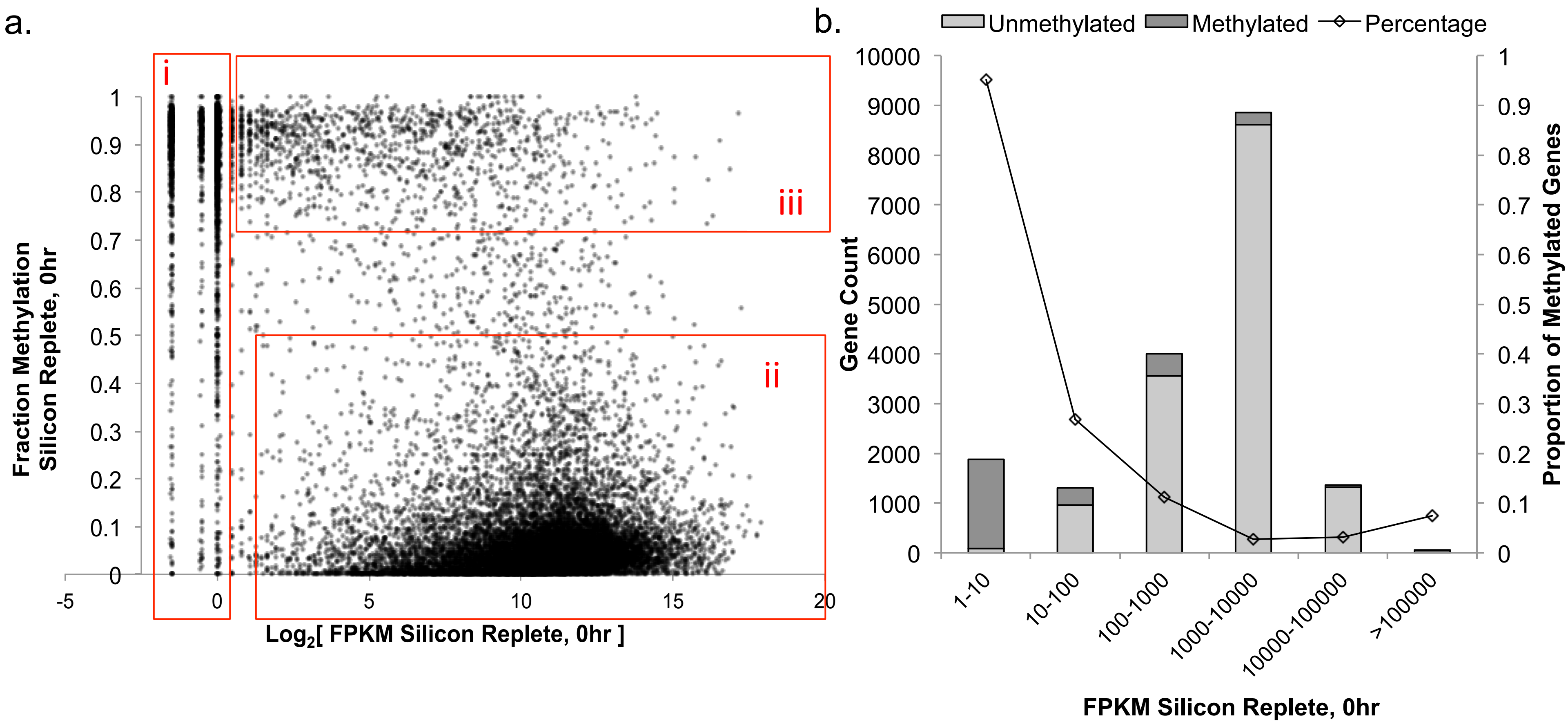

Figure S8: Vector map for rpL41 construct for *C. cryptica.*

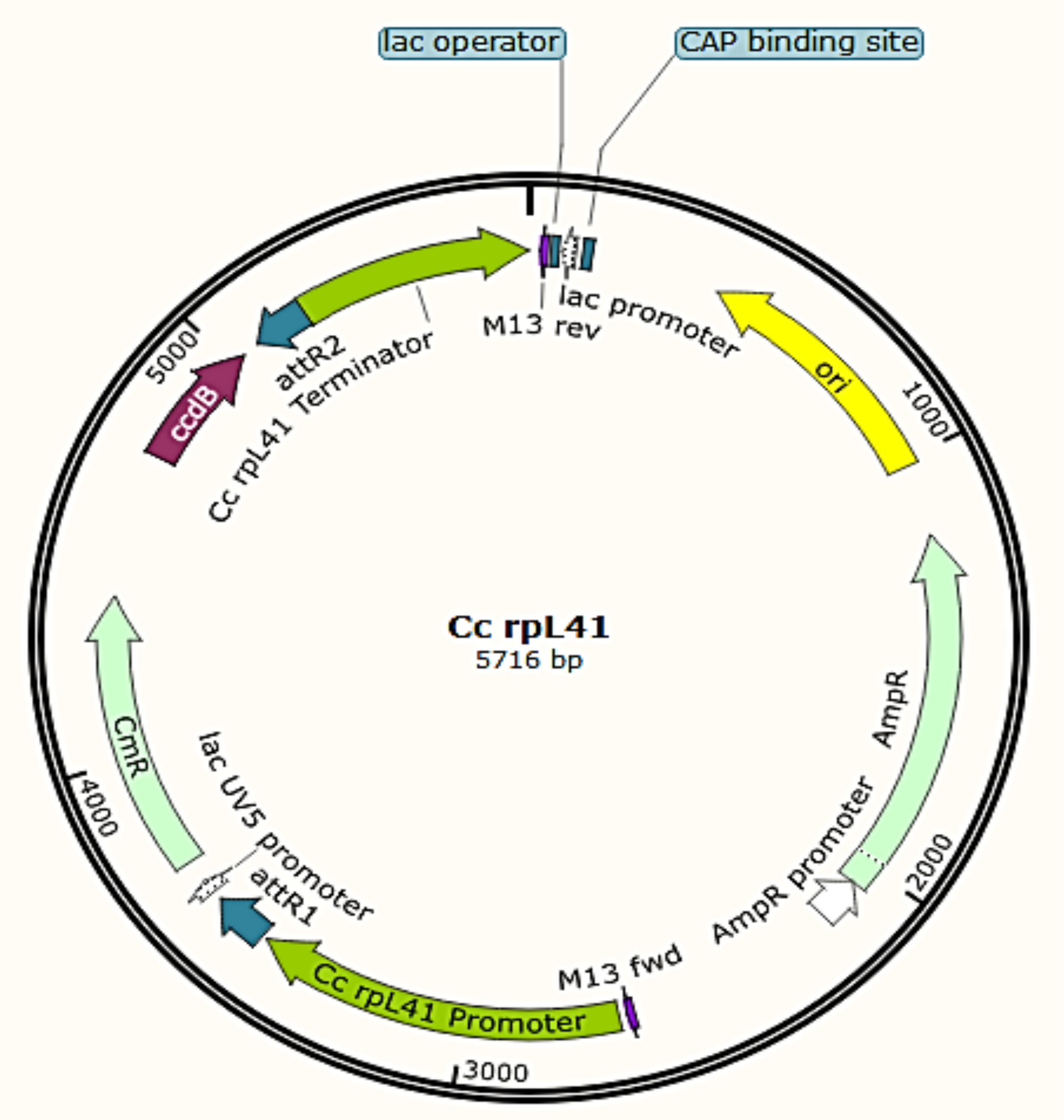

Supplement: Supplementary file 1 — Additional file 1. Additional methods and figures. Figure S1. (a) Cyclotella cryptica nuclear assembly overview broken down by minimum contig size (Kbp) versus total Mbp of all contigs (left) and minimum contig size versus the total number of contigs (right) and (b) genomic sequencing data. Table S1. Statistics from different gene model prediction pipelines. The pipelines are: (1) FGENESH gene predictions, Diatom training set, (2) Augustus gene predictions V1, Chlamydomonas training set, no RNAseq data, (3) Augustus gene predictions V2, FGENESH100 training set, RNAseq data (4) Augustus gene predictions V3, ‘self’ trained, RNAseq data (5) MAKER gene predictions, (Augustus self trained + FGENESH + GeneMarkES). Details are presented in Additional methods. Data presented includes all predicted models regardless of read counts from RNAseq data and prior to removing apparent duplicate contigs (Additional methods, Genome Library Construction and Sequencing). Table S2. Repeat sequences in C. cryptica identified using (a) RepBase data and (b) RepeatModeler data. Figure S2. Phylogenetic comparison of diatom species with sequenced genomes. (a) 18S sequence comparison from [91], accession numbers are listed in Additional file 4. (b) Reciprocal best blast hits comparison. C. cryptica and T. pseudonana are the most similarly related centric diatoms with available genomic data. Figure S3. Per-cytosine fraction methylation. (a) 0 h, silicon replete sample (b) 48 h, silicon-deplete sample. All sites shown have a read coverage greater than or equal to 4. Figure S4. Per-site methylation differences between silicon replete and deplete conditions. (a) Histogram showing absolute value of the difference between 48 and 0 h. (b) Scatter plot showing the slope between fraction methylation of genes when comparing the two conditions is linear, outliers are apparent. Figure S5. Fraction methylation across the eight largest contigs in C. cryptica. Blue is 0 h silicon replete sample, red is 48 h [file 13068_2016_670_MOESM1_ESM.pdf]
